# Association Between Sleep Traits and Rheumatoid Arthritis: A Mendelian Randomization Study

**DOI:** 10.3389/fpubh.2022.940161

**Published:** 2022-06-30

**Authors:** Rui-Chen Gao, Ni Sang, Cheng-Zhen Jia, Meng-Yao Zhang, Bo-Han Li, Meng Wei, Guo-Cui Wu

**Affiliations:** School of Nursing, Anhui Medical University, Hefei, China

**Keywords:** autoimmune disease, rheumatoid arthritis, Mendelian randomization, sleep, causality

## Abstract

Currently, the causal association between sleep disorders and rheumatoid arthritis (RA) has been poorly understood. In this two-sample Mendelian randomization (TSMR) study, we tried to explore whether sleep disorders are causally associated with RA. Seven sleep-related traits were chosen from the published Genome-Wide Association Study (GWAS): short sleep duration, frequent insomnia, any insomnia, sleep duration, getting up, morningness (early-to-bed/up habit), and snoring, 27, 53, 57, 57, 70, 274, and 42 individual single-nucleotide polymorphisms (SNPs) (*P* < 5 × 10^−8^) were obtained as instrumental variables (IVs) for these sleep-related traits. Outcome variables were obtained from a public GWAS study that included 14,361 cases and 43,923 European Ancestry controls. The causal relationship between sleep disturbances and RA risk were evaluated by a two-sample Mendelian randomization (MR) analysis using inverse variance weighted (IVW), MR-Egger regression, weighted median, and weight mode methods. MR-Egger Regression and Mendelian randomization pleiotropy residual sum and outlier (MR-PRESSO) were used to test for horizontal pleomorphism and outliers. There was no evidence of a link between RA and frequent insomnia (IVW, odds ratio (OR): 0.99; 95% interval (CI): 0.84–1.16; *P* = 0.858), any insomnia (IVW, OR: 1.09; 95% CI: 0.85–1.42; *P* = 0.489), sleep duration (IVW, OR: 0.65, 95% CI: 0.38–1.10, *P* = 0.269), getting up (IVW, OR: 0.56, 95% CI: 0.13–2.46, *P* = 0.442), morningness (IVW, OR: 2.59; 95% CI: 0.73–9.16; *P* = 0.142), or snoring (IVW, OR: 0.95; 95% CI: 0.68–1.33; *P* = 0.757). Short sleep duration (6h) had a causal effect on RA, as supported by IVW and weighted median (OR: 1.47, 95% CI: 1.12–1.94, *P* = 0.006; OR: 1.43, 95%CI:1.01–2.05, *P* = 0.047). Sensitivity analysis showed that the results were stable. Our findings imply that short sleep duration is causally linked to an increased risk of RA. Therefore, sleep length should be considered in disease models, and physicians should advise people to avoid short sleep duration practices to lower the risk of RA.

## Introduction

Rheumatoid arthritis (RA) is a chronic inflammatory immune system disease that affects joint synovial tissue, tendon sheaths, and bursa, with symptoms of joint pain, stiffness, joint swelling, deformity, functional disability, and sleep disturbances (1). The global prevalence of RA is estimated to be around 1%, with a lower prevalence in some countries. The incidence rises with age, with women over 65 having the highest rate. RA Patients have a higher burden of mortality and morbidity, as well as a lower life quality and a higher disability rate (2). Many patients were given disease-modifying antirheumatic drugs (DMARDs) do not achieve effective treatment or develop resistance (3, 4). The actual pathophysiological mechanisms of RA are unknown, though studies have suggested that Interactions between genes and the environment, immune dysfunctions, and interstitial tissue disorders may all play a role (3). As a result, it is critical that more efforts be made to investigate the etiology of RA in order to promote treatment strategies with minimal or no side effects.

Sleep disturbances are a common but unrecognized feature of rheumatism (5). Sleep has a significant and broad impact on human immunity, is critical to human health, and is increasingly recognized as a significant lifestyle choice, with studies reporting that decreased sleep duration is associated with an increased risk of cerebrovascular disease and death in the general public (6). An animal model study conducted in New Zealand suggests that sleep deprivation causes immune disorders that may include not only a reduced response to pathogens, but also destroys immune self-tolerance which promotes the emergence of autoimmune diseases (7). According to studies, 50–75% of RA patients suffer from sleep disturbances (8), poor sleep quality and decreased total sleep time are common complaints in RA patients (9, 10), insomnia affects more than half of all patients (11).

Sleep disorders have been linked to RA in cross-sectional studies, but the findings are conflicting (12–14), it is unknown whether sleep problems cause or are independent risk factors for RA. According to data from a baseline assessment of cohort studies of sleep disorders in RA revealed that 87.4% of people had short sleep duration. As disease activity worsens, physical health conditions include pain, fatigue, and dysfunction are impacted, and sleep disorders become more common and future studies will need to look into a causal relationship between poor sleep quality and disease activity (15–17). After controlling for variables, the baseline data from the UK Biobank cohort reveal that rheumatoid factor level (RF) status was still linked with sleep duration, implying that sleep length may influence RF (18). These results show that sleep disturbances may contribute to the development of rheumatoid arthritis. However, whether sleep disturbances are causally associated with RA has rarely been investigated, and conducting clinical trials to answer this question is very difficult.

Mendelian randomization study is an epidemiological study design and data analysis method based on Mendelian's law of independent distribution to verify the etiology hypothesis. It has been widely utilized to evaluate environmental risk variables and illness etiology (19). MR adopts germline genetic variants as proxies. Because environmental factors and disease processes have no effect on genetic variants, MR can reduce the influence of other factors, enhance the exposure-outcome relationship, and remove reverse causality (20). It is thought to be a reliable method for circumventing the limits of observational studies by employing genetic variation as an instrumental variable and large-scale data from GWASs (21). In this study, we aimed to conduct a two-sample MR study to estimate the causal relationship between sleep traits and RA.

## Materials and Methods

### Data Sources

Genetic information is used as an IV in Mendelian randomization studies (19). We carried out a two-sample MR study using data based on publicly available GWAS. Preceding published SNPS associated with each sleep phenotype were chosen as IVs (Supplementary Table 1).

#### Short Sleep

Park et al. (22) used genetic instruments (genetic variants associated with short sleep behavior as instrumental variables) to conduct a MR analysis on 321260 White British individuals and defined short sleep as <6 h. To investigate sleep duration, a standardized touchscreen questionnaire was used: “How much sleep do you get every 24 hours?” (Naps should be included). Sleep time was collected on an hourly basis, and was classified as short (<6 h), medium (6–8 h), and long (≥9 h) groups. Data from the UK Biobank prospective cohort were studied, patients aged 40–69 years, contain 25,605 self-reported short duration sleep (6 h per 24 h), 404,550 reported intermediate sleep (6–8 h), and 35,659 reported long sleep (9 h). MR analysis was performed on 321,260 White British people in the clinical study. As a result, 27 SNPs were associated with short sleep duration and 8 SNPs with long sleep duration. We did not perform MR analysis on long sleep because there were insufficient SNPs in the long sleep data.

#### Insomnia Symptoms

Participants of European descent in the UK Biobank (*n* = 453,379) self-reported insomnia symptoms by answering the question “Do you have difficulty falling asleep at night or wake up in the middle of the night”. In this sample, 29% of individuals self-reported frequent insomnia symptoms (usually), 57 loci for self-reported insomnia symptoms were discovered (23). The study performed two parallel GWAS: (1) Frequent insomnia symptoms: participants whose responses occurred frequently were defined as the case group, while those whose responses never or rarely occurred were defined as the control group (*n* = 129,270 patients) and 108,357 controls); (2) Any symptoms of insomnia: participants whose responses were sometimes or usually frequent were defined as cases, while those whose responses never or rarely appeared were defined as controls (*n* = 345,022 and 108,357 controls), resulting in 57 SNPs associated with frequent insomnia and any symptoms of insomnia.

#### Sleep Duration

Jansen et al. (24) obtained a sample size of 13,31,010 individuals by combining data from the UK Biobank (UKB) version 2 (*n* = 386,533) and 23 and Me, a privately held personal genomics and biotechnology company (*n* = 944,477). Jansen et al. assessed sleep duration by asking, “About how many hours sleep do you get in every 24 hours?”. Only integer values could be used in the answer (round hours). The average sleep duration per 24 h was 7.10 h. The sleep duration GWAS analysis discovered 3,886 GWS SNPs (*P* < 5 × 10^−8^) and 53 SNPS were found to be related to sleep duration which were mapped to 49 independent genomic loci. Sleep duration was analyzed as a continuous outcome.

#### Ease of Getting up in the Morning

Jansen et al. (24) assessed the ease of getting up in the morning in a GWAS study of 1,331,010 people by answering, “In general, how easy do you find getting up in the morning?” There were four possible responses: “not at all easy,” “not very easy,” “fairly easy,” and “very easy.” The ease of getting out of bed in the morning was examined as a continuous outcome, divided into four categories. The ease of getting up GWAS analysis revealed 7248 GWS SNPs (*P* < 5 × 10^−8^), finally, 70 SNPs associated with ease of getting up were discovered, which were mapped to 62 independent genomic loci.

#### Morningness

In the GWAS study involving 1,331,010 individuals by Jansen et al. (24), the following responses were given to the question “Do you consider yourself to be?” “Definitely a ‘morning’ person,” “More a ‘morning’ than an ‘evening’ person,” “More an ‘evening’ than a ‘morning’ person,” “Definitely an ‘evening’ person,” and “Do not know,” and was analyzed on a continuous scale. The morningness GWAS analysis discovered 16,805 GWS SNPs (*P* < 5 × 10^−8^), and 274 SNPs associated with morningness were discovered and mapped to 207 independent genomic loci.

#### Snoring

The snoring GWAS analysis discovered 3416 GWS SNPs (*P* < 5 × 10^−8^), represented by 42 independent lead SNPs that were mapped to 36 distinct genomic loci. Snoring was evaluated by asking the question, “Does your partner, a close relative, or a friend complain about your snoring?” Participants could respond with a “yes” or a “no.” Finally, 42 snoring-related SNPs were discovered (24).

#### RA

The GWAS information about RA were acquired from a public GWAS website (https://gwas.mrcieu.ac.uk/), ID: ieu—a−832, the study from the European people involved in 14,361 cases and 43,923 controls and 87,47,963 SNPs. Okada Y et al. published the results of a GWAS meta-analysis of RA related loci in this database in 2014 (25).

### SNP Selection

We used the following steps to select the IVs to ensure that genetic instruments were associated with sleep. First, as IVs, SNPs that were significantly associated with sleep were chosen. A set of SNPS that fell below the genome-wide statistical significance threshold (*P* < 5 × 10^−8^) was used. Second, for meaningful SNPs, the minor allele frequency (MAF) threshold was set at 0.01. Thirdly, in this study, the clumping process (*R*^2^ < 0.001, clumping distance = 10,000 kb) was used to evaluate the linkage disequilibrium (LD) between the contained SNPs. Fourth, the effect of SNPs on exposure should correspond to the same allele as the effect on outcome. Palindromic SNPs are removed from instrumental variables. Fifth, if no exposure-related SNPs were found in the results, proxy SNPs that were significantly associated with meaningful variables (*R*^2^ > 0.8) were chosen.

### The Assumptions of MR

Two sample MR must meet three assumptions: that genetic instrumental variables are associated with the exposure (relevance assumption), that F statistics are usually employed to evaluate the intensity of the correlation between instrumental variables and exposures. The equation for the F statistic is F = *R*^2^(n-k-1)/k(1-*R*^2^). *R*^2^ denotes the exposure variance of the chosen SNPs interpretation, n is the sample size, and K represents the number of instrumental variables included. If F is <10, there is a weak relationship between the IVs and exposure (26); that they are unrelated to confounding factors (independence assumption); and that they influence the outcome solely through their effects on the exposure of interest (exclusion restriction assumption), implying that there is no horizontal pleiotropic effect between IV and outcomes (27).

### MR Estimates

Four different methods were used to assess the causal effects of RA outcomes: Weighted median, MR-Egger regression, IVW, weighted mode (28). When each genetic variant meets the assumptions of an instrumental variable, IVW methods provide consistent estimates of the causal effect of exposure on outcomes (29). Egger's method and weighted median methods provide consistent causal estimates of multiple genetic variants from pooled data under weaker assumptions (28). Even when up to 50% of the information comes from the genetic variation of invalid IV, weighted median estimates still yield consistent estimates of causal effects. The weighted median estimate maintains a more accurate estimate than the MR-Egger analysis (30). The MR-Egger regression methods and MR-PRESSO analysis were adopted to identify and adjust for pleiotropy. The MR-Egger regression analysis, which is robust to invalid instruments, tests and explain the presence of unbalanced pleiotropy by mergering summary data estimates of causal effects from multiple individual variants (31). MR-Egger employs the weighted linear regression of the gene-outcome coefficients on the gene-exposure coefficients. Estimates of causal effects are expressed by the slope of linear regression, and estimates of the mean horizontal pleiotropic effects of genetic variation are shown by intercept (32). Heterogeneity between estimates for each SNP was evaluated using Cochran's Q statistic (33). Sensitivity analysis was performed by eave-one-out Sensitivity test to assess the stability of effect size and identify single SNPS that had a disproportionate impact on association (34). All statistical analyses were performed using R version 4.0.1 with the Two Sample MR 0.5.6 package. The statistical significance was double-tailed, *P* < 0.05.

## Results

Supplementary Table 1 shows the data sources. The detailed information of the IVs is provided in Supplementary Tables S2–S8. The details of the SNPS included in MR analysis are shown in Supplementary Table S9. The visualization results of MR analysis are shown in Supplementary Figures S1–S6.

### Short Sleep Duration and RA

**Figure 1:**
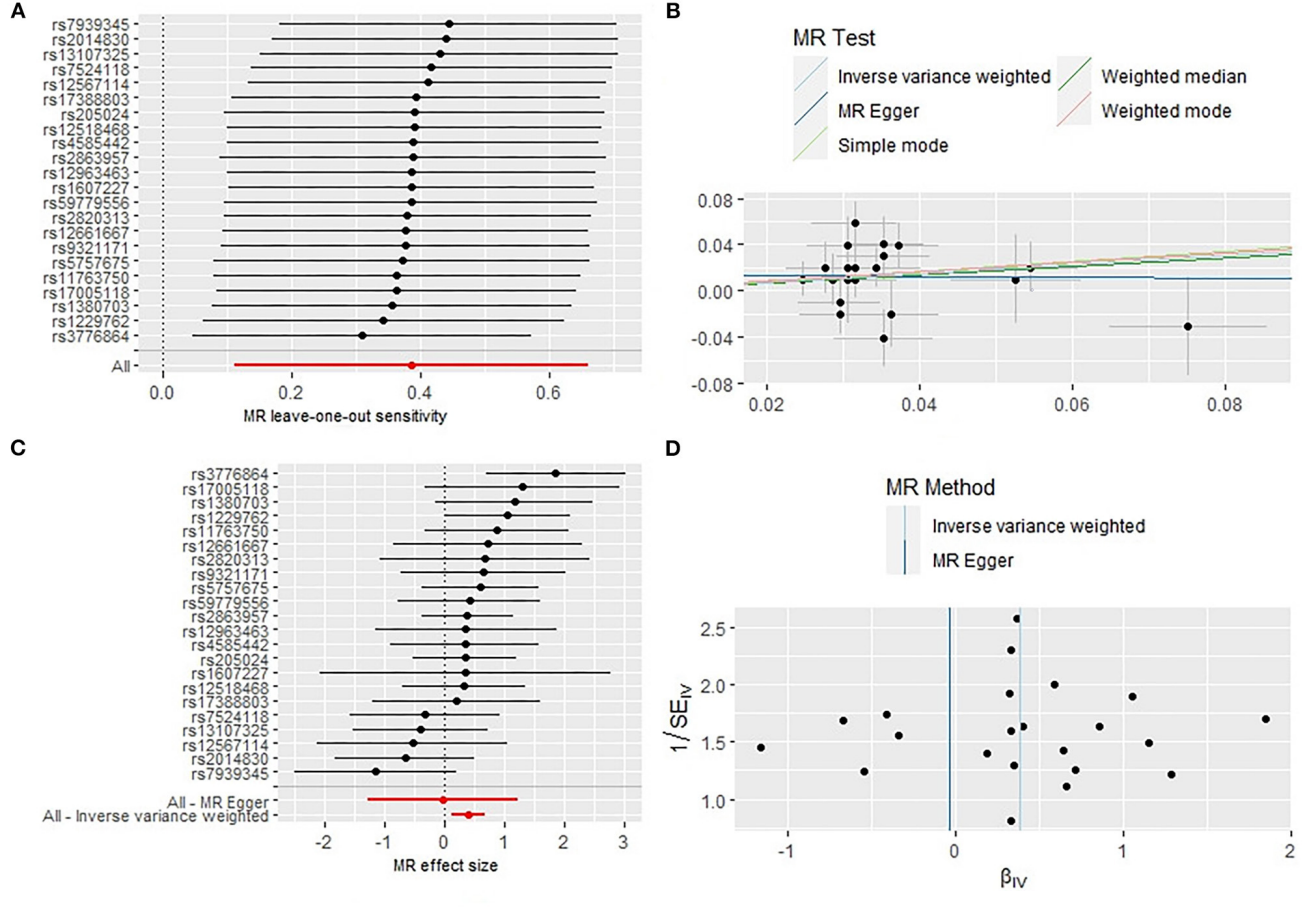
Sensitivity analysis **(A)**, scatter plot **(B)**, forest plot **(C)**, and funnel plot **(D)** of the causal effect of short sleep duration on RA risk.

Initially a total of 27 genome-wide significant SNPs were identified as IVs from the Mendelian Randomization Study performed by Park et al. (22). However, due to the LD with other SNPs, two SNPs (rs75539574 and rs142180737) were removed. SNPs that not associated with RA were removed, the effect alleles were aligned, and all SNPs with palindromic structures were removed, finally, 22 SNPS were included in the next two-sample MR analysis, explaining 0.29% of the variance of short sleep (<6 h). We also collected SNP effect allele, other allele, EAF, beta, se and *P*-value. The results of IVW analyses and weighted median demonstrated that short sleep (<6 h) were positively correlated with the risk of RA (OR: 1.47,95%CI: 1.12–1.94, *P* = 0.005; OR: 1.43, 95%CI: 1.01–2.05, *P* = 0.047) (Table 1) (Figure 1C). The MR-Egger regression method failed to identify the horizontal pleomorphism between IV and outcomes (Table 1) (Figure 1B). However, the MR-PRESSO method found horizontal pleiotropy (*P* = 0.031), and rs60882754 was identified as outlier. Results did not change after removing abnormal SNPs (IVW, β = 0.39, 95% CI, 0.11–0.66, *P* = 0.006). No heterogeneity was detected (*Q* = 23.65, *P* = 0.258) (Figure 1D). The results of the leave-one-out sensitivity test showed that the results of the MR analysis were robust (Figure 1A). The detailed information of the IV was shown in Supplementary Table S2. The *F* statistics of the SNPs was >10, indicating that there was no weak instrumental variables bias (Table 1).

**Table 1:** MR results of causal links between sleep traits and RA risk.

**Exposure**	**N SNP**	**Methods**	**β (95% CI)**	**OR (95%CI)**	**SE**	***P* value**	**Horizontal pleiotropy**							**Heterogeneity**			
							**MR-Egger**				**MR-**			**Cochran's**	***P***	P^*^	***F***
							**regression**				**PRESSO**			***Q***	**value**	***value***	
							**Egger**	**SE**	***P***	**Global test**	**Outliers**	β^*^	P^*^				
							**intercept**		**value**	***P* value**		**(95% CI)**	***value***				
Short sleep	22	MR Egger	−0.04 (−1.28–1.21)	0.96 (0.28–3.36)	0.64	0.954	0.01	0.02	0.503	0.031	rs60882754	−0.04 (−1.28–1.21)	0.954	23.65	0.258	0.258	42.65
		Weighted median	0.36 (0.01–0.72)	1.43 (1.01–2.05)	0.18	0.047						0.36 (−0.00–0.72)	0.068				
		Inverse variance weighted	0.39 (0.11–0.66)	1.47 (1.12–1.94)	0.14	0.006						0.39 (0.11–0.66)	0.006				
		Weighted mode	0.40 (−0.16–0.96)	1.50 (0.86–2.62)	0.29	0.172						0.40 (−0.14–0.14)	0.179				
Frequent insomnia	32	MR Egger	0.12 (−0.38–0.62)	1.13 (0.68–1.85)	0.25	0.645	0.01	0.01	0.583	0.457				28.67	0.535		51.25
		Weighted median	−0.14 (−0.37 0.10)	0.87 (0.69–1.10)	0.12	0.249											
		Inverse variance weighted	−0.01 (−0.17–0.35)	0.99 (0.84–1.16)	0.08	0.858											
		Weighted mode	−0.12 (−0.43 0.18)	0.88 (0.65–1.20)	0.15	0.430											
Any insomnia	33	MR Egger	0.01 (−0.73–0.76)	1.01 (0.48–2.14)	0.38	0.974	0.00	0.01	0.829	0.041	rs10156602	0.01 (−0.74–0.77)	0.972	46.52	0.036	0.028	39.93
		Weighted median	0.08 (−0.24–0.40)	1.08 (0.78 1.50)	0.16	0.625						0.06 (−0.27–0.39)	0.715				
		Inverse variance weighted	0.09 (−0.17–0.35)	1.09 (0.85–1.42)	0.13	0.489						0.09 (−0.18–0.35)	0.515				
		Weighted mode	0.01 (−0.39–0.41)	1.01 (0.67–1.51)	0.21	0.964						−0.03 (−0.42–0.36)	0.862				
Sleep duration	30	MR Egger	−1.02 (−2.36–0.32)	0.36 (0.09–1.37)	0.68	0.684	0.01	0.01	0.354	0.027	rs12215241	−0.87 (−2.06–0.32)	0.163	42.53	0.039	0.233	36.32
		Weighted median	−0.5 (−1.17–0.18)	0.61 (0.31–1.19)	0.34	0.344						−0.5 (−1.18–0.19)	0.157				
		Inverse variance weighted	−0.43 (−0.96–0.10)	0.65 (0.38–1.10)	0.27	0.269						−0.3 (−0.77 0.17)	0.215				
		Weighted mode	−0.66 (−1.54–0.22)	0.52 (0.21–1.24)	0.45	0.448						−0.67 (−1.49 0.16)	0.124				
Getting up	43	MR Egger	1.46 (−2.47–5.38)	4.29 (0.08–216.94)	2.00	0.471	0.03	0.03	0.278	<5e-04	rs2193749, rs553108, rs61773374	0.16 (−1.37 1.69)	0.838	368.25	0.000	0.165	28.72
		Weighted median	0.22 (−0.62–1.05)	1.24 (0.54–2.85)	0.43	0.612						−0.29 (−1.11–0.52)	0.477				
		Inverse variance weighted	−0.58 (−2.06–0.90)	0.56 (0.13–2.46)	0.76	0.442						−0.23 (−0.80–0.35)	0.440				
		Weighted mode	−0.09 (−1.19–1.00)	0.91 (0.30–2.72)	0.56	0.867						−0.30 (−1.30–0.70)	0.566				
Morningness	109	MR Egger	0.95 (−0.31 2.21)	2.59 (0.73–9.16)	0.64	0.142	0.02	0.01	0.099	<0.0003	rs486416, rs6716898, rs9295795	0.68 (−0.07–1.43)	0.078	422.25	0.000	0.008	47.92
		Weighted median	0.30 (−0.10–0.70)	1.36 (0.91–2.02)	0.20	0.136						0.30 (−0.09–0.69)	0.126				
		Inverse variance weighted	−0.05 (−0.51 0.41)	0.95 (0.60–1.51)	0.23	0.837						0.12 (−0.15–0.40)	0.385				
		Weighted mode	−0.24 (−0.84–0.36)	0.78 (0.43–1.43)	0.31	0.427						−0.27 (−0.92–0.37)	0.405				
Snoring	22	MR Egger	1.24 (−0.41–2.88)	3.44 (0.67–17.77)	0.838	0.156	0.04	0.03	0.132	0.109				28.33	0.102		49.26
		Weighted median	0.27 (−0.11–0.66)	1.32 (0.90–1.93)	0.196	0.163											
		Inverse variance weighted	−0.05 (−0.39–0.28)	0.95 (0.68–1.33)	0.171	0.757											
		Weighted mode	0.3 (−0.30–0.89)	1.35 (0.74–2.434)	0.303	0.336											

* *The result of recalculation after removing outliers*. *>MR, Mendelian Randomization; MR-PRESSO, MR-Pleiotropy Residual Sum and Outlier method; OR, odds ratio; CI, confidence interval; IVW, inverse-variance weighted*.

### Frequent Insomnia and RA

A total of 57 SNPs were associated with frequent insomnia in the study by Lane et al. (23). 43 SNPs remained after removal of LD. After removing SNPs with independence from RA, aligning effect allele, and removing all SNPs with palindromic, the remaining 40 SNPs were finally included in the next two-sample MR analysis, explaining 0.68% of the variance of frequent insomnia. The results of IVW analyses demonstrated no association between frequent insomnia and RA (OR, 0.99; 95% CI, 0.84–1.16; *P* = 0.858) (Table 1) (Supplementary Figure S1C). Horizontal pleiotropy between instrumental variables and outcomes was not identified by the MR-Egger regression (Table 1) (Supplementary Figure S1B) method and the MR-PRESSO method (*P* = 0.583; *P* = 0.457) (Table 1), and no heterogeneity was detected (*Q* = 28.67, *P* = 0.570) (Table 1) (Supplementary Figure S1D). The detailed information of the IV was shown in Supplementary Table S3. There was no weak instrumental variables bias (*F* = 51.25).

### Any Insomnia and RA

In the study of Lane et al. (23) a total of 57 SNPs were related to sleep difficulty, and 43 SNPs remained after removing the LD. After removing SNPs independent of RA, and aligning the effect allele, 40 SNPs remained, and 33 SNPs were included in the next two-sample MR analysis, explaining 0.29% of the variance of any insomnia. The results of IVW analyses did not find any insomnia to be associated with RA (OR, 1.09; 95% CI, 0.85–1.42; *P* = 0.489) (Table 1) (Supplementary Figure S2C). The MR-Egger regression method did not identify horizontal pleiotropy between IV and outcomes (Table 1) (Supplementary Figure S2B). The heterogeneity test showed the existence of heterogeneity (*Q* = 46.52, *P* = 0.036) (Table 1) (Supplementary Figure S2D). The MR-PRESSO method detected the outlier rs10156602. After removing the outlier, MR analysis showed no significant change (IVW, β = 0.09, 95% CI, −0.18–0.35, *P* = 0.515) the heterogeneity still existed (*P* = 0.028) (Table 1). The detailed information of the IV was shown in Supplementary Table S4. There was no weak instrumental variables bias (*F* = 39.93).

### Sleep Duration and RA

We selected 53 SNPs in the GWAS of Jansen et al. (24) as IVs. 10 SNPs were deleted due to LD (rs1392817, rs11682175, rs2863244, rs11883686, rs34388845, rs1633063, rs6979198, rs7778250, rs1668331, rs62061734). SNPs that independent of RA were removed, and the effect allele was aligned and all SNPs with palindromic structures were removed. The last 30 SNPs were included in the next two-sample MR analysis, explaining 0.08% of the variance of sleep duration. MR analysis showed that sleep duration was not associated with RA (OR: 0.65, 95% CI: 0.38–1.10, *P* = 0.269) (Table 1) (Supplementary Figure S3C). The MR-Egger regression method did not identify horizontal pleiotropy between instrumental variables and outcomes (*P* = 0.354) (Table 1) (Supplementary Figure S3B). The MR-PRESSO method found horizontal pleiotropy (*P* = 0.027), and rs12215241 was identified as outlier, and rs12215241 was identified as outlier. However, the results did not change when the abnormal SNP values were removed (IVW, β = −0.3, 95% CI, −0.77–0.17, *P* = 0.215) (Table 1). Heterogeneity was detected between SNPs (Q = 42.53, *P* = 0.039) (Table 1) (Supplementary Figure S3D). The heterogeneity disappeared after removing outliers (*P* = 0.233). Supplementary Table S5 displays detailed IV information. The SNPs had F-statistics >10, indicating that there was no weak instrumental variable bias (Table 1).

### Getting up and RA

We selected 70 SNPs from the GWAS of Jansen et al. (24), 17 SNPs were removed due to LD (rs148173313, rs6691053, rs75650221, rs4652514, rs1402121, rs10180284, rs13393656, rs35333999, rs4483990, rs11520042, rs17464772, rs1017168, rs61963491, rs1949072, rs7222039, rs3746601, rs4634827). After removing SNPs with independence from RA, performing effect allele alignment and removing all SNPs with palindromic, 43 SNPs were included in the next two-sample MR analysis, explaining 0.09% of the variance of getting up. MR analysis showed that there was no causal relationship between getting up and RA (IVW, OR: 0.56, 95% CI: 0.13–2.46, *P* = 0.442) (Table 1) (Supplementary Figure S4C). The MR-Egger regression method did not identify horizontal pleiotropy between instrumental variables and outcomes (*P* = 0.278) (Table 1) (Supplementary Figure S4B) MR-PRESSO test found 3 outliers (rs2193749, rs553108, rs61773374), MR analysis was performed after removing outliers, and the results did not change significantly (IVW, β = −0.23, 95% CI: −0.80–0.35, *P* = 0.440) (Table 1). The detailed information of the IV was shown in Supplementary Table S6. No heterogeneity was found after removing outliers (*P* = 0.18) (Table 1). There was no weak instrumental variables bias (*F* = 28.72) Table 1.

### Morningness and RA

In the study of Jansen et al. (24), 274 SNPs were associated with morning phenotype, and 131 SNPs were removed due to LD, after removing the SNPs independent of RA, 131 SNPs remained after aligning the alleles. Finally, the remaining 109 SNPs were included in the MR analysis, explaining 0.39% of the variance of the morningness type, The results of IVW analyses indicated no association between the morningness type and RA (OR: 2.59; 95% CI: 0.73–9.16; *P* = 0.142) (Table 1) (Supplementary Figure S5C). The MR-Egger regression method did not identify horizontal pleiotropy between IV and outcomes (*P* = 0.099) (Table 1) (Supplementary Figure S5B). The MR-PRESSO method detected outliers rs486416, rs6716898, and rs9295795. Heterogeneity test was performed to find heterogeneity among SNPs (Table 1) (Supplementary Figure S5D). After removing the outliers, the results did not change, and the heterogeneity still existed (*P* = 0.008) (Table 1). The specific information of the IV was displayed in Supplementary Table S7. There was no weak instrumental variables bias (*F* = 47.92).

### Snoring and RA

There were 42 SNPs related to snoring in the study of Jansen et al. (24), 11 SNPs were removed due to LD (rs35915391, rs10190879, rs745558, rs2762049, rs2408111, rs7217107, rs4792897, rs1563304, rs2924251, rs60, rs2924251, rs60, rs2924251, rs64). After SNPs that were independent of RA were removed, 28 SNPs remained after allele alignment and removal of the SNPS with palindromic structure. The last 22 SNPs were included in the MR analysis, explaining 0.08% of the variance in snoring. The results of IVW analyses did not find association between snoring and RA (OR: 0.95; 95% CI: 0.68–1.33; *P* = 0.757) (Table 1) (Supplementary Figure S6C). The MR-Egger regression (Table 1) (Supplementary Figure S6B) and MR-PRESSO method did not identify horizontal pleiotropy between instrumental variables and outcomes (*P* = 0.132; *P* = 0.109) (Table 1). There was no heterogeneity in the results (*P* = 0.102) (Supplementary Figure S6D). The detailed information of the IV was shown in Supplementary Table S8. There was no weak instrumental variables bias (*F* = 49.26).

## Discussion

In this TSMR study, we investigated the effects of seven sleep traits on RA, including short sleep, frequent insomnia, any insomnia, sleep duration, getting up, morningness, and snoring. Short sleep duration (<6 h) was linked to an increased risk of RA. However, because fewer IVs reached the genome-wide statistical significance threshold, the results and accuracy of sleep may have been affected.

RA is a chronic inflammatory autoimmune disease, the pathogenesis of which has not been fully clarified. It may be associated with inflammation, synovial cells and cartilage cells, autoantibodies, genetics, immune responses. Immune disturbances can have an effect on sleep quality, and sleep disturbances can also have an impact on immune function. Sleep is especially important for triggering an effective adaptive immune response, which results in long-term immune memory (35). T_reg_ inhibitory activity was reduced in healthy sleep-deprived subjects. T_regs_ play a crucial role in suppressing inappropriate immune responses and preserving self-tolerance. The disruption of self-tolerance is nuclear to the pathogenesis of most autoimmune diseases, establishing a linkage between sleep disorders and autoimmune diseases (36). Sleep disturbances cause an increase in several pro-inflammatory cytokines, and IL-17 remains elevated despite recovering within 7 days of sleep deprivation; IL-17 plays a key role in coordinating inflammation; and IL-17 is linked to the onset of RA (37). Sleep disorders can impair immune defenses and cause inflammatory changes throughout the body, triggering the onset of RA.

However, our findings revealed that frequent insomnia, any insomnia, sleep duration, getting up, morningness, and snoring has nothing to do with an increased risk of RA. According to data from a large, prospective study in Norway, after controlling for confounding factors, there is evidence that insomnia increases the risk of rheumatoid arthritis (38). A longitudinal study found that pain in RA patients predicted sleep problems 2 years later, but that sleep problems had no effect on subsequent pain (39). Several cross-sectional studies have also linked sleep problems to pain (2), depression (40), disease activity, and fatigue (41). These findings suggest that sleep disturbance may be an adverse consequence of RA, which may be related to joint pain, activity limitation, and medication in active disease, which cannot fully explain that sleep disturbance is the cause of RA. Since the immune system appears to play a significant function in regular sleep, variation in inflammatory load can influence sleep quality, and cytokines such as interleukin (IL)-1β, IL-6, or tumor necrosis factor (TNF) have been found to alter animals and play a key role in human sleep quality (42), Elevated levels of IL-6 are associated with sleep disorders and fatigue symptoms in inflammatory diseases such as RA, and insufficient sleep time and quality can also stimulate the elevation of IL-6 levels and promote the progression of RA(43), these inflammatory factors are closely related to the pathogenesis of RA(44). This suggests that sleep disorders do not directly influence the incidence of RA, but they may influence the incidence of RA via inflammatory mediators.

In recent years, more and more observational evidence has shown that short sleep duration is a risk factor for the progress of RA. A cohort study found that short sleepers (≤6 h/day) were 1.23 times more possible to develop RA than those who slept 7–8 h (95% CI 1.101–1.51), after adjusting for confounding factors (17). An observational study showed that RA patients do have sleep disorders, especially short sleep duration. Poor sleep quality and short sleep duration may be the inducement or risk factor of RA (45). However, most of these previous studies were cross-sectional, retrospective or prospective cohort studies and, due to their observational nature, could not overcome the influence of unmeasured confounding factors on the results. MR methods were adopted to research the causal effect of genetically predicted short sleep duration on RA risk, which may control for unmeasured confounders and reverse causality. The complex interactions between the central nervous system, endocrine system, and immune system play an important role in normal sleep, and sleep problems in RA patients may arise at various levels of these systems. Although sleep disturbances are more common in RA patients, studies have shown that sleep disturbances do not change with improvement in disease activity (46). The Swedish “RA Epidemiological Survey” from 1998 to 2018 collected sleep data from 1 to 12 years after diagnosis and did not find any major sleep problems. The existing sleep problems were mainly related to pain and functional decline (47). This may explain to a certain extent that short sleep time may be the cause of RA, and there is a one-way causal relationship between short sleep time and RA. Our findings also suggest a causal effect between short sleep duration and RA, suggesting that shorter sleep duration may increase the risk of RA.

Our study has the following advantages: First, as far as we know, this is the first study to investigate the genetic link between sleep traits and RA risk, and while some previous cross-sectional studies have controlled for confounding factors, there may still be undetected biases. Second, because of the large number of published genetic associations used to screen for suitable genetic instrumental variables, MR analysis is a time and cost-effective method for assessing and screening potential causal relationships. Based on large sample sizes, we were capable to investigate the effect of short sleep, sleep duration, frequent insomnia, any insomnia, morningness, getting up, and snoring on RA using a two-sample MR design (14,361 cases and 43,923 controls). This broadens the scope of our findings. Third, in the MR analysis, genes are used as IV, and the possibility of these genes being related to environmental confounders is very small. Genotypes are randomly distributed during pregnancy, prior to exposure, and the association between genotypes and disease is not affected by reverse causality. Exposure-related genotypes are typically linked from birth to adulthood, avoiding attenuation due to causal inference error (regression dilution bias).

Our study has several limitations that should be considered. First, because not all genes that determine all traits are isolated independently, Mendel's second law does not apply to all genetic variants (randomly) and genes as IVs cannot avoid bias due to weak instrumental variable, population stratification, and developmental compensation. MR genetic instruments are chosen from hypothesis-free genome-wide association studies, which may lack a thorough understanding of the mechanisms underlying associations between genetic variants and diseases (48), this could have an impact on the results. Second, our study is limited to the European population. As a result, it is necessary to conduct additional studies to determine whether our study can be extended to other populations. Third, all sleep characteristics were described subjectively by the patient. As a result, misclassification is unavoidable. Fourth, even if we took steps to identify and eliminate outlier variants, we cannot rule out the possibility that horizontal pleiotropy influenced our results. Finally, in our MR analysis, each method has advantages and disadvantages. However, we used four methods based on different assumptions, which may result in inconsistent results, thereby clouding the study's conclusions.

## Conclusion

Our results reveal the causal relationship between gene-predicted sleep traits and RA, and we only identify the causal relationship between short sleep and RA, which is somewhat inconsistent with many published observational studies. To verify our results more accurately, updated MR analyses are necessary when more advanced methods yield better accuracy or GWAS aggregate data, and more RA patient information becomes available.

## Data Availability Statement

The original contributions presented in the study are included in the article/Supplementary Material, further inquiries can be directed to the corresponding author.

## Author Contributions

G-CW designed this study. R-CG and NS analyzed data, and wrote the draft of the manuscript. R-CG, NS, C-ZJ, M-YZ, B-HL, and MW discussed and reviewed the manuscript critically. G-CW and R-CG revised the manuscript. All authors contributed to the article and approved the submitted version.

## Funding

This work was supported by the Anhui Provincial Natural Science Foundation (1908085MH294); the scientific research cultivation project for School of Nursing, Anhui Medical University (hlqn2020007); and the Research Fund of Anhui Institute of Translational Medicine (2021zhyx-C28).

## Conflict of Interest

The authors declare that the research was conducted in the absence of any commercial or financial relationships that could be construed as a potential conflict of interest.

## Publisher's Note

All claims expressed in this article are solely those of the authors and do not necessarily represent those of their affiliated organizations, or those of the publisher, the editors and the reviewers. Any product that may be evaluated in this article, or claim that may be made by its manufacturer, is not guaranteed or endorsed by the publisher.
