# Supplementary material for: Association Between Sleep Traits and Rheumatoid Arthritis: A Mendelian Randomization Study
Source: Front Public Health. 2022 Jun 30;10:940161. doi: 10.3389/fpubh.2022.940161 (PMC9280285; doi:10.3389/fpubh.2022.940161)

Supplementary material

Table S1. Characteristics of sleep traits in UK Biobank and rheumatoid arthritis consortium.

Phenotype	Type of trait	Author, published year	Consortium	Sample size	No. of cases (Binary trait)	PMID
Short sleep duration	Binary	Park, S. et al, 2020	UKB	321260	25,605	33004418
Frequent Insomnia	Binary	Lane, J. M. et al, 2019	UKB	237627	129,270	30804566
Any Insomnia	Binary	Lane, J. M. et al, 2019	UKB	453379	345,022	30804566
Sleep duration	continuous	Jansen, P. R.2019	UKB and 23andMe	1331010	NA	30804565
Getting up	continuous	Jansen, P. R.2019	UKB and 23andMe	1331010	NA	30804565
Morningness	continuous	Jansen, P. R.2019	UKB and 23andMe	1331010	NA	30804565
snoring	Binary	Jansen, P. R.2019	UKB and 23andMe	1331010	359,916	30804565
Rheumatoid arthritis	Binary	Okada Y. 2014	NA	58,284	14,361	24390342

Table S2. The summary information for instrumental variables of short sleep duration.

phenotype	SNP	effect_ allele	other_ allele	eaf	P value	beta	se	Sample size
Short sleep duration	rs2863957	C	A	0.782	2.60E-18	0.054488185	0.006243162	321260
Short sleep duration	rs13107325	T	C	0.075	2.50E-13	0.075107472	0.010262117	321260
Short sleep duration	rs1229762	T	C	0.665	1.10E-12	0.037295785	0.005240102	321260
Short sleep duration	rs1380703	G	A	0.384	1.60E-11	0.035367144	0.005248498	321260
Short sleep duration	rs12963463	C	T	0.299	1.90E-11	0.028587457	0.004258199	321260
Short sleep duration	rs75539574	A	C	0.915	8.40E-11	0.044973366	0.006926164	321260
Short sleep duration	rs17388803	C	A	0.106	6.50E-10	0.05259245	0.008513132	321260
Short sleep duration	rs4585442	G	A	0.311	8.10E-10	0.030529205	0.004969791	321260
Short sleep duration	rs1607227	G	T	0.705	1.50E-09	0.030529205	0.005050854	321260
Short sleep duration	rs2820313	G	A	0.341	2.30E-09	0.030529205	0.005109436	321260
Short sleep duration	rs17005118	A	G	0.265	2.50E-09	0.030529205	0.005121099	321260
Short sleep duration	rs5757675	G	T	0.26	2.70E-09	0.034401427	0.005782851	321260
Short sleep duration	rs12567114	G	A	0.725	4.10E-09	0.036331929	0.006178787	321260
Short sleep	rs142180737	C	T	0.009	4.40E-09	0.154436353	0.026316568	321260

duration								
Short sleep duration	rs2186122	T	A	0.562	4.80E-09	0.023716527	0.004051364	321260
Short sleep duration	rs11763750	G	A	0.814	5.10E-09	0.035367144	0.006052001	321260
Short sleep duration	rs12518468	C	T	0.328	8.50E-09	0.031498667	0.005470199	321260
Short sleep duration	rs9367621	T	A	0.431	1.60E-08	0.023716527	0.004197258	321260
Short sleep duration	rs3776864	A	C	0.667	1.70E-08	0.031498667	0.00558482	321260
Short sleep duration	rs60882754	A	T	0.939	1.80E-08	0.055434707	0.009845956	321260
Short sleep duration	rs59779556	T	G	0.554	2.00E-08	0.024692613	0.004399966	321260
Short sleep duration	rs2014830	C	T	0.698	2.70E-08	0.029558802	0.005316471	321260
Short sleep duration	rs205024	C	T	0.617	2.70E-08	0.030529205	0.005491008	321260
Short sleep duration	rs12661667	T	C	0.263	2.80E-08	0.027615167	0.004972567	321260
Short sleep duration	rs7939345	T	G	0.208	4.00E-08	0.035367144	0.006441103	321260
Short sleep duration	rs9321171	C	T	0.54	4.20E-08	0.031498667	0.005745595	321260
Short sleep duration	rs7524118	C	T	0.708	4.90E-08	0.029558802	0.00541876	321260

Table S3. The summary information for instrumental variables of frequent insomnia.

phenotype	SNP	effect_allele	other_allele	eaf	pval	beta	se	Sample size
frequent insomnia	rs11184946	T	C	0.417223	2.90E-10	0.012837	0.003147	237627
frequent insomnia	rs6664467	G	A	0.863468	4.50E-08	0.021189	0.003117	237627
frequent insomnia	rs4751	T	G	0.425368	1.60E-08	0.012837	0.003147	237627
frequent insomnia	rs2644128	G	C	0.549876	1.00E-12	0.017033	0.003117	237627
frequent insomnia	rs12405761	A	C	0.570608	2.60E-11	0.017033	0.002088	237627
frequent insomnia	rs11804386	A	G	0.332415	2.60E-08	0.017033	0.003117	237627
frequent insomnia	rs62158170	A	G	0.783643	5.70E-13	0.021189	0.004098	237627
frequent insomnia	rs4577309	A	G	0.468107	3.70E-09	0.012837	0.003147	237627
frequent insomnia	rs35881094	G	T	0.426213	3.00E-15	0.021189	0.003117	237627
frequent insomnia	rs113851554	T	G	0.057694	1.30E-41	0.079181	0.007241	237627
frequent insomnia	rs72826719	A	G	0.049281	2.20E-12	0.041393	0.00688	237627
frequent insomnia	rs9845387	C	A	0.959308	2.10E-08	0.041393	0.0079	237627
frequent insomnia	rs4683301	T	A	0.599949	4.60E-08	0.017033	0.003147	237627
frequent insomnia	rs10865954	T	C	0.333944	1.70E-09	0.017033	0.003147	237627
frequent insomnia	rs4688760	T	C	0.690062	1.00E-12	0.021189	0.003117	237627
frequent insomnia	rs11097861	G	A	0.715424	1.70E-09	0.017033	0.002088	237627
frequent insomnia	rs1841625	G	A	0.431882	3.60E-08	0.012837	0.003178	237627
frequent insomnia	rs28061	A	G	0.692042	2.10E-08	0.017033	0.003147	237627
frequent insomnia	rs1592757	C	G	0.356945	4.60E-10	0.017033	0.003117	237627
frequent insomnia	rs7711696	T	G	0.304975	9.90E-12	0.021189	0.003117	237627

frequent insomnia	rs1430205	T	C	0.458038	3.60E-08	0.012837	0.003147	237627
frequent insomnia	rs6932158	C	T	0.490999	2.80E-08	0.012837	0.003147	237627
frequent insomnia	rs314280	G	A	0.54824	3.50E-10	0.01703	0.00315	237627
frequent insomnia	rs10947690	G	A	0.261294	3.50E-08	0.017033	0.004176	237627
frequent insomnia	rs3824081	T	C	0.474743	1.10E-08	0.017033	0.003117	237627
frequent insomnia	rs10280045	G	C	0.573651	1.00E-10	0.017033	0.002088	237627
frequent insomnia	rs302165	G	A	0.216374	2.40E-06	0.017033	0.004176	237627
frequent insomnia	rs6593005	G	A	0.740998	8.60E-09	0.012837	0.003147	237627
frequent insomnia	rs17151854	T	G	0.153806	2.40E-08	0.025306	0.004098	237627
frequent insomnia	rs10156602	A	G	0.638126	3.40E-12	0.017033	0.002088	237627
frequent insomnia	rs2296580	G	T	0.702115	8.70E-12	0.021189	0.004098	237627
frequent insomnia	rs11191595	A	C	0.936734	1.50E-09	0.041393	0.0079	237627
frequent insomnia	rs10838708	G	A	0.541245	2.50E-09	0.012837	0.002108	237627
frequent insomnia	rs68094047	T	C	0.250411	3.30E-09	0.017033	0.003117	237627
frequent insomnia	rs324017	A	C	0.294759	1.10E-10	0.021189	0.003088	237627
frequent insomnia	rs2956278	G	A	0.214809	1.30E-08	0.021189	0.004137	237627
frequent insomnia	rs1923770	T	A	0.383028	2.30E-12	0.021189	0.003117	237627
frequent insomnia	rs1031654	C	A	0.201527	2.00E-09	0.021189	0.004137	237627
frequent insomnia	rs4886140	G	A	0.668277	3.10E-10	0.017033	0.003117	237627
frequent insomnia	rs11635495	C	T	0.514746	6.80E-09	0.017033	0.003147	237627
frequent insomnia	rs4886860	G	C	0.23362	6.10E-12	0.02119	0.00312	237627
frequent insomnia	rs1544637	T	C	0.487502	3.00E-08	0.012837	0.002108	237627

frequent insomnia	rs3104778	A	G	0.589387	4.20E-08	0.012837	0.003178	237627
frequent insomnia	rs2062113	T	C	0.429502	1.90E-10	0.017033	0.003147	237627
frequent insomnia	rs17139246	C	T	0.389356	4.10E-08	0.012837	0.003147	237627
frequent insomnia	rs17669584	G	A	0.19491	3.60E-08	0.01703	0.00312	237627
frequent insomnia	rs11651809	G	C	0.296	2.10E-15	0.021	0.003	237627
frequent insomnia	rs1942262	A	G	0.292258	1.10E-13	0.017033	0.003117	237627
frequent insomnia	rs11673344	G	A	0.380184	9.00E-10	0.017033	0.003117	237627

Table S4. The summary information for instrumental variables of any insomnia.

phenotype	SNP	effect_ allele	other_ allele	eaf	pval	Sample size	beta	se
Any insomnia	rs11184946	T	C	0.417223	2.00E-07	453379	0.019802627	0.003808683
Any insomnia	rs6664467	G	A	0.863468	7.60E-06	453379	0.029558802	0.006603617
Any insomnia	rs4751	T	G	0.425368	6.30E-05	453379	0.019802627	0.00494907
Any insomnia	rs2644128	G	C	0.549876	2.90E-11	453379	0.029558802	0.004443889
Any insomnia	rs12405761	A	C	0.570608	4.40E-08	453379	0.029558802	0.005399859
Any insomnia	rs2613503	A	C	0.801866	4.90E-08	453379	0.029558802	0.00541876
Any insomnia	rs11804386	A	G	0.332415	7.00E-09	453379	0.029558802	0.005104332
Any insomnia	rs62158170	A	G	0.783643	1.30E-16	453379	0.048790164	0.005897107
Any insomnia	rs4577309	A	G	0.468107	2.10E-08	453379	0.029558802	0.005275009
Any insomnia	rs12713372	C	T	0.565758	8.50E-09	453379	0.029558802	0.005133313
Any insomnia	rs35881094	G	T	0.426213	8.50E-13	453379	0.039220713	0.005483236
Any insomnia	rs2192338	C	G	0.779652	5.90E-09	453379	0.039220713	0.006739455
Any insomnia	rs113851554	T	G	0.057694	2.10E-28	453379	0.131028262	0.011853626
Any insomnia	rs72826719	A	G	0.049281	3.80E-06	453379	0.058268908	0.012606774
Any insomnia	rs9845387	C	A	0.959308	1.00E-06	453379	0.067658648	0.01383149
Any insomnia	rs6785034	A	G	0.42201	3.40E-08	453379	0.029558802	0.005355358
Any insomnia	rs4683301	T	A	0.599949	1.80E-05	453379	0.019802627	0.004617766
Any insomnia	rs10865954	T	C	0.333944	4.70E-09	453379	0.029558802	0.005046352
Any insomnia	rs4688760	T	C	0.690062	7.30E-12	453379	0.039220713	0.005724268
Any insomnia	rs55946513	C	T	0.93413	1.40E-08	453379	0.048790164	0.008599827
Any insomnia	rs11097861	G	A	0.715424	4.00E-09	453379	0.029558802	0.005023425
Any insomnia	rs1841625	G	A	0.431882	1.00E-05	453379	0.019802627	0.004483099
Any insomnia	rs28061	A	G	0.692042	3.40E-07	453379	0.029558802	0.005796063
Any insomnia	rs1592757	C	G	0.356945	2.80E-05	453379	0.019802627	0.004727122
Any insomnia	rs7711696	T	G	0.304975	3.10E-07	453379	0.029558802	0.005776291
Any insomnia	rs1430205	T	C	0.458038	7.60E-08	453379	0.029558802	0.005497905
Any insomnia	rs6932158	C	T	0.490999	1.30E-05	453379	0.029558802	0.006779378
Any insomnia	rs314280	G	A	0.54824	0.00041	453379	0.019802627	0.005604155
Any insomnia	rs10947690	G	A	0.261294	9.30E-07	453379	0.029558802	0.006025154
Any insomnia	rs3824081	T	C	0.474743	2.50E-10	453379	0.039220713	0.006198959
Any insomnia	rs10280045	G	C	0.573651	3.80E-06	453379	0.029558802	0.006395197
Any insomnia	rs302165	G	A	0.216374	3.30E-08	453379	0.029558802	0.005350275
Any insomnia	rs6593005	G	A	0.740998	1.30E-06	453379	0.019802627	0.00409166
Any insomnia	rs17151854	T	G	0.153806	8.00E-05	453379	0.029558802	0.007493865
Any insomnia	rs11793831	G	T	0.583428	2.10E-08	453379	0.029558802	0.005275009
Any insomnia	rs11793074	A	G	0.853086	8.00E-09	453379	0.039220713	0.006799167
Any insomnia	rs10156602	A	G	0.638126	1.20E-08	453379	0.029558802	0.005186004
Any insomnia	rs2296580	G	T	0.702115	2.20E-08	453379	0.039220713	0.007009344
Any insomnia	rs11191595	A	C	0.936734	2.10E-09	453379	0.076961041	0.012848518
Any insomnia	rs10838708	G	A	0.541245	1.40E-05	453379	0.019802627	0.004558762

Any insomnia	rs68094047	T	C	0.250411	2.20E-07	453379	0.029558802	0.005704579
Any insomnia	rs324017	A	C	0.294759	2.20E-08	453379	0.029558802	0.005282612
Any insomnia	rs2956278	G	A	0.214809	2.50E-07	453379	0.029558802	0.005731003
Any insomnia	rs2147141	G	C	0.543173	5.30E-09	453379	0.029558802	0.005063634
Any insomnia	rs1923770	T	A	0.383028	4.70E-09	453379	0.029558802	0.005046352
Any insomnia	rs1031654	C	A	0.201527	6.50E-07	453379	0.039220713	0.007882393
Any insomnia	rs4886140	G	A	0.668277	9.90E-07	453379	0.029558802	0.006040278
Any insomnia	rs11635495	C	T	0.514746	3.60E-06	453379	0.019802627	0.004274041
Any insomnia	rs4886860	G	C	0.23362	1.30E-10	453379	0.039220713	0.006102329
Any insomnia	rs1544637	T	C	0.487502	9.80E-06	453379	0.019802627	0.004478672
Any insomnia	rs3104778	A	G	0.589387	2.50E-07	453379	0.019802627	0.003839429
Any insomnia	rs2062113	T	C	0.429502	3.40E-07	453379	0.029558802	0.005796063
Any insomnia	rs17139246	C	T	0.389356	1.30E-05	453379	0.019802627	0.004541778
Any insomnia	rs17669584	G	A	0.19491	5.00E-08	453379	0.039220713	0.007194731
Any insomnia	rs11651809	G	C	0.296	5.00E-12	453379	0.039220713	0.005679571
Any insomnia	rs1942262	A	G	0.292258	3.10E-11	453379	0.029558802	0.00445046
Any insomnia	rs11673344	G	A	0.380184	7.10E-08	453379	0.029558802	0.00548541

Table S5. The summary information for instrumental variables of sleep duration.

phenotype	SNP	effect_allele	other_allele	beta	se	eaf	pval	samplesize
Sleep duration	rs4642942	C	G	0.01	0.002492	0.4148	3.07E-05	1331010
Sleep duration	rs915416	C	G	0.02	0.002694	0.2906	8.15E-12	1331010
Sleep duration	rs540431	G	A	-0.01	0.002465	0.4502	4.91E-06	1331010
Sleep duration	rs1392817	A	G	0.01	0.002493	0.4773	1.84E-05	1331010
Sleep duration	rs79512144	A	G	-0.03	0.004972	0.0648	3.64E-05	1331010
Sleep duration	rs11682175	C	T	0.02	0.002457	0.4734	1.09E-10	1331010
Sleep duration	rs116219610	T	C	0.02	0.00311	0.1927	4.75E-11	1331010
Sleep duration	rs2863244	G	A	-0.01	0.002491	0.4382	3.18E-05	1331010
Sleep duration	rs62158206	C	T	0.04	0.002968	0.2189	3.00E-40	1331010
Sleep duration	rs35662245	A	T	0.02	0.002582	0.3389	6.23E-06	1331010
Sleep duration	rs11883686	T	A	-0.01	0.002601	0.3322	1.01E-05	1331010
Sleep duration	rs12463754	G	C	0.02	0.002771	0.2706	3.91E-07	1331010
Sleep duration	rs112230981	G	A	-0.03	0.005698	0.0485	4.59E-05	1331010
Sleep duration	rs4688116	G	T	-0.01	0.002508	0.4033	4.20E-05	1331010
Sleep duration	rs16834426	A	G	0.02	0.002763	0.2685	1.76E-05	1331010
Sleep duration	rs9843801	C	T	0.02	0.002584	0.3414	5.82E-10	1331010
Sleep duration	rs2192528	A	G	0.02	0.002452	0.4772	6.27E-07	1331010
Sleep duration	rs7686205	G	A	0.01	0.002519	0.3794	1.37E-05	1331010
Sleep duration	rs41501452	G	A	-0.02	0.002633	0.3155	4.04E-06	1331010
Sleep duration	rs17285646	A	T	0.01	0.002452	0.4764	1.88E-05	1331010
Sleep duration	rs13109404	G	T	-0.03	0.004795	0.0704	1.13E-08	1331010
Sleep duration	rs365663	G	A	-0.02	0.002462	0.4551	1.12E-08	1331010
Sleep duration	rs6889592	A	G	0.02	0.002596	0.3324	1.44E-08	1331010
Sleep duration	rs113322698	A	G	0.01	0.002574	0.3424	1.94E-05	1331010
Sleep duration	rs12215241	A	G	-0.02	0.002931	0.2247	6.09E-10	1331010
Sleep duration	rs34388845	G	A	-0.02	0.002983	0.2136	5.43E-07	1331010
Sleep duration	rs1633063	T	C	-0.02	0.002839	0.246	3.17E-06	1331010
Sleep duration	rs9451146	T	C	-0.02	0.002915	0.228	8.53E-06	1331010
Sleep duration	rs9362971	C	T	-0.01	0.002567	0.3461	1.41E-05	1331010
Sleep duration	rs3823624	C	T	0.02	0.003188	0.1807	1.30E-06	1331010
Sleep duration	rs6979198	G	T	0.02	0.003	0.2109	1.07E-05	1331010
Sleep duration	rs7778250	C	A	-0.01	0.002696	0.2898	4.96E-05	1331010
Sleep duration	rs1668331	T	G	-0.02	0.00254	0.3738	5.20E-07	1331010
Sleep duration	rs651902	C	T	0.01	0.002534	0.3843	3.35E-09	1331010
Sleep duration	rs12336359	C	G	0.01	0.002523	0.4037	9.42E-06	1331010
Sleep duration	rs10973207	T	G	0.02	0.003391	0.1549	2.02E-06	1331010
Sleep duration	rs144625846	G	A	-0.02	0.003575	0.1346	1.05E-05	1331010
Sleep duration	rs7915425	T	C	0.02	0.003221	0.1758	3.08E-07	1331010
Sleep duration	rs7115856	C	A	0.01	0.002452	0.4613	1.25E-06	1331010
Sleep duration	rs12791153	T	A	0.03	0.004614	0.0772	2.89E-05	1331010
Sleep duration	rs1553132	G	A	0.02	0.002788	0.2586	6.63E-06	1331010

Sleep duration	rs1263056	G	A	-0.01	0.002471	0.4817	4.15E-05	1331010
Sleep duration	rs4767550	G	A	0.01	0.002507	0.4128	2.70E-06	1331010
Sleep duration	rs11621908	T	C	-0.03	0.004548	0.0796	4.95E-06	1331010
Sleep duration	rs13329140	A	G	-0.02	0.002888	0.2359	7.51E-08	1331010
Sleep duration	rs77684884	G	A	0.02	0.003197	0.1878	5.83E-07	1331010
Sleep duration	rs28651105	G	A	0.02	0.002969	0.2165	4.75E-05	1331010
Sleep duration	rs8047587	T	G	-0.02	0.002468	0.4396	1.09E-09	1331010
Sleep duration	rs11076146	G	T	0.01	0.002461	0.44	2.96E-06	1331010
Sleep duration	rs11654671	T	A	0.02	0.002928	0.2348	3.71E-05	1331010
Sleep duration	rs11650677	A	G	0.01	0.00258	0.3394	8.95E-09	1331010
Sleep duration	rs62061734	C	T	-0.02	0.002967	0.2205	2.17E-06	1331010
Sleep duration	rs12607679	C	T	-0.02	0.002821	0.2595	9.79E-08	1331010

Table S6. The summary information for instrumental variables of getting up.

phenotype	SNP	effect_allele	other_allele	beta	se	eaf	pval	samplesize
Getting up	rs61773374	G	A	0.02	0.0022	0.195	3.37E-14	1331010
Getting up	rs148173313	A	G	0.02	0.0022	0.0055	9.39E-08	1331010
Getting up	rs301806	C	T	-0.01	0.0017	0.4208	4.73E-05	1331010
Getting up	rs77576965	T	C	0.01	0.0019	0.2727	1.94E-05	1331010
Getting up	rs12752290	C	T	0.01	0.0017	0.4421	1.02E-09	1331010
Getting up	rs113240734	A	G	0.02	0.0023	0.1601	2.17E-13	1331010
Getting up	rs148137538	G	A	0.04	0.0045	0.0231	1.48E-08	1331010
Getting up	rs6691053	T	C	0.04	0.0045	0.2144	3.43E-05	1331010
Getting up	rs75650221	T	C	0.04	0.0045	0.0381	3.93E-15	1331010
Getting up	rs4652514	C	T	-0.01	0.0019	0.3018	9.91E-06	1331010
Getting up	rs12736689	C	T	0.05	0.0051	0.0297	1.66E-21	1331010
Getting up	rs76048411	T	C	0.01	0.0017	0.4833	8.53E-07	1331010
Getting up	rs2053457	C	T	-0.02	0.002	0.2465	1.29E-12	1331010
Getting up	rs1402121	C	T	0.01	0.0022	0.1998	6.66E-08	1331010
Getting up	rs10180284	T	C	0.01	0.0022	0.4834	4.91E-05	1331010
Getting up	rs4671328	T	G	0.01	0.0018	0.4478	1.06E-06	1331010
Getting up	rs10175975	T	C	0.01	0.0023	0.179	3.30E-06	1331010
Getting up	rs13393656	A	C	0.01	0.002	0.2426	2.10E-05	1331010
Getting up	rs406952	C	T	0.01	0.0018	0.3765	1.20E-05	1331010
Getting up	rs4853283	G	A	-0.01	0.0017	0.4401	1.84E-13	1331010
Getting up	rs1606803	T	C	0.01	0.0019	0.2871	3.22E-07	1331010
Getting up	rs35333999	T	C	-0.02	0.0024	0.043	3.44E-06	1331010
Getting up	rs116298301	T	C	-0.02	0.0024	0.0257	2.40E-11	1331010
Getting up	rs4483990	C	A	-0.02	0.0024	0.1549	6.40E-10	1331010
Getting up	rs13116306	T	C	-0.01	0.0018	0.42	1.76E-05	1331010
Getting up	rs10470887	G	A	-0.01	0.0017	0.4874	4.05E-05	1331010
Getting up	rs9995419	A	G	0.01	0.002	0.2369	1.50E-05	1331010
Getting up	rs79751662	C	G	-0.02	0.0028	0.1067	2.32E-05	1331010
Getting up	rs1459192	T	C	-0.01	0.0018	0.3288	2.50E-05	1331010
Getting up	rs12515274	A	G	-0.01	0.002	0.2523	5.37E-07	1331010
Getting up	rs4958316	A	C	0.02	0.0019	0.2883	3.69E-12	1331010
Getting up	rs553108	A	G	0.01	0.0018	0.4159	3.12E-05	1331010
Getting up	rs2653349	A	G	0.02	0.0021	0.213	8.45E-26	1331010
Getting up	rs9399613	T	C	-0.01	0.0019	0.2861	1.32E-05	1331010
Getting up	rs3735478	T	G	-0.01	0.0019	0.2908	1.57E-05	1331010
Getting up	rs2944822	T	C	0.01	0.0017	0.4452	4.43E-06	1331010
Getting up	rs16917522	C	T	0.01	0.0022	0.1827	1.12E-06	1331010
Getting up	rs72663537	G	T	-0.01	0.0026	0.1295	2.76E-05	1331010
Getting up	rs77641763	T	C	-0.02	0.0026	0.122	1.98E-08	1331010
Getting up	rs4962716	T	C	-0.02	0.0026	0.1258	1.82E-06	1331010
Getting up	rs11229264	A	G	-0.01	0.0019	0.2982	1.43E-07	1331010

Getting up	rs7297799	T	C	-0.01	0.0018	0.3989	1.21E-09	1331010
Getting up	rs11520042	C	T	0.01	0.0018	0.3455	7.90E-06	1331010
Getting up	rs17464772	A	G	0.01	0.0018	0.3508	1.03E-08	1331010
Getting up	rs2193749	T	C	-0.01	0.0017	0.4947	1.84E-05	1331010
Getting up	rs6581138	A	G	0.01	0.002	0.2434	2.30E-07	1331010
Getting up	rs1017168	A	C	-0.01	0.0018	0.3551	2.64E-05	1331010
Getting up	rs74643199	T	A	-0.01	0.0024	0.1538	1.25E-05	1331010
Getting up	rs4884166	A	G	-0.01	0.0022	0.1984	1.15E-05	1331010
Getting up	rs7332608	G	A	-0.03	0.0048	0.0338	4.55E-08	1331010
Getting up	rs61963491	G	A	-0.03	0.0048	0.0058	5.13E-06	1331010
Getting up	rs6575012	A	G	-0.01	0.0017	0.4819	2.57E-05	1331010
Getting up	rs3935182	G	C	0.01	0.0018	0.4207	6.77E-09	1331010
Getting up	rs1420607	A	G	0.01	0.0019	0.2713	1.57E-08	1331010
Getting up	rs11642015	T	C	0.01	0.0018	0.4041	6.20E-08	1331010
Getting up	rs1949072	A	G	0.01	0.0017	0.4342	2.48E-05	1331010
Getting up	rs17822102	G	A	0.01	0.0018	0.3437	9.40E-07	1331010
Getting up	rs11643192	A	C	-0.01	0.0018	0.3893	1.30E-06	1331010
Getting up	rs4790352	G	A	-0.02	0.0031	0.0812	8.78E-06	1331010
Getting up	rs3760185	T	C	-0.01	0.002	0.2433	4.52E-05	1331010
Getting up	rs7222039	T	C	0.01	0.0017	0.4467	3.42E-05	1331010
Getting up	rs12150229	G	A	0.01	0.0021	0.2236	3.52E-06	1331010
Getting up	rs77556405	A	G	0.02	0.0023	0.1727	1.59E-10	1331010
Getting up	rs12601968	T	G	-0.01	0.0018	0.3261	1.16E-06	1331010
Getting up	rs4395148	T	A	-0.01	0.0019	0.308	2.36E-05	1331010
Getting up	rs8182491	T	C	-0.02	0.0029	0.1005	1.18E-06	1331010
Getting up	rs3746601	C	A	0.01	0.0018	0.3603	2.85E-05	1331010
Getting up	rs11697690	C	T	0.01	0.0017	0.469	2.33E-05	1331010
Getting up	rs74555583	A	G	-0.02	0.0031	0.0855	3.04E-05	1331010
Getting up	rs4634827	G	A	0.01	0.0021	0.3412	9.12E-07	1331010

Table S7. The summary information for instrumental variables of morningness.

phenotype	SNP	effect_allele	other_allele	beta	se	eaf	pval	samplesize
Morningness	rs150812083	C	G	-0.153	0.016	0.9954	4.06E-19	1331010
Morningness	rs61773390	T	G	0.029	0.003	0.1955	2.75E-24	1331010
Morningness	rs228654	A	G	-0.03	0.004	0.0975	2.65E-13	1331010
Morningness	rs12065331	T	C	-0.015	0.002	0.3068	1.51E-07	1331010
Morningness	rs17448682	T	C	0.018	0.003	0.2315	4.04E-10	1331010
Morningness	rs7543480	T	C	0.016	0.002	0.3447	6.90E-10	1331010
Morningness	rs10916866	A	C	-0.017	0.002	0.5887	2.39E-11	1331010
Morningness	rs12140153	T	G	-0.027	0.004	0.0877	4.62E-09	1331010
Morningness	rs11208844	A	G	-0.017	0.003	0.1379	2.81E-05	1331010
Morningness	rs11162296	C	G	0.036	0.003	0.16	1.50E-31	1331010
Morningness	rs10873936	A	G	-0.013	0.002	0.3098	9.04E-06	1331010
Morningness	rs17416934	T	C	0.013	0.002	0.616	5,93E-09	1331010
Morningness	rs74802342	A	G	-0.019	0.003	0.1125	2.09E-05	1331010
Morningness	rs72720396	A	G	-0.022	0.003	0.7706	3.29E-15	1331010
Morningness	rs7522677	T	C	0.015	0.003	0.2099	7.91E-06	1331010
Morningness	rs9437742	C	G	-0.012	0.002	0.526	3.53E-05	1331010
Morningness	rs12139650	T	G	0.015	0.003	0.8131	2.21E-05	1331010
Morningness	rs10494041	C	G	0.02	0.003	0.8269	1.01E-09	1331010
Morningness	rs35461065	T	C	-0.012	0.002	0.5397	8.36E-06	1331010
Morningness	rs956796	A	G	-0.019	0.003	0.1092	2.42E-05	1331010
Morningness	rs12401456	A	G	-0.018	0.003	0.1667	7.98E-07	1331010
Morningness	rs2794682	T	C	0.021	0.002	0.396	3.93E-19	1331010
Morningness	rs2055975	A	C	0.016	0.002	0.2895	3.95E-09	1331010
Morningness	rs148137538	A	G	-0.041	0.007	0.9769	6.46E-06	1331010
Morningness	rs75650221	T	C	0.034	0.006	0.0381	8,92E-10	1331010
Morningness	rs13306728	A	G	0.03	0.004	0.9239	6.16E-11	1331010
Morningness	rs7534480	T	C	0.017	0.002	0.385	2.87E-12	1331010
Morningness	rs509476	T	C	0.099	0.006	0.03	7.41E-53	1331010
Morningness	rs12746073	T	C	0.012	0.002	0.5259	1.73E-05	1331010
Morningness	rs12025393	A	G	-0.014	0.002	0.2653	1.82E-05	1331010
Morningness	rs16839841	T	G	0.022	0.004	0.9188	2.69E-05	1331010
Morningness	rs13011556	C	G	-0.016	0.003	0.7622	7.73E-08	1331010
Morningness	rs2712056	T	C	0.018	0.003	0.1845	1.57E-07	1331010
Morningness	rs848552	C	G	-0.013	0.002	0.4724	1.11E-06	1331010
Morningness	rs62135536	T	C	0.035	0.006	0.0313	1.41E-05	1331010
Morningness	rs2592199	C	G	0.022	0.003	0.7693	1.35E-11	1331010
Morningness	rs10495976	A	T	-0.017	0.002	0.6114	1.01E-10	1331010
Morningness	rs10173945	T	G	0.013	0.002	0.6185	2.71E-06	1331010
Morningness	rs72831198	T	C	-0.016	0.003	0.8235	4.68E-05	1331010
Morningness	rs1520524	T	C	0.025	0.004	0.1006	2.70E-09	1331010
Morningness	rs10193431	T	C	0.013	0.002	0.5255	4.35E-06	1331010

Morningness	rs17049270	T	C	-0.02	0.004	0.0959	1.96E-05	1331010
Morningness	rs10175975	T	C	0.018	0.003	0.179	1.94E-07	1331010
Morningness	rs359237	T	C	-0.015	0.002	0.4678	9.18E-09	1331010
Morningness	rs4672440	T	G	0.016	0.002	0.3465	5.09E-10	1331010
Morningness	rs113851554	T	G	-0.028	0.005	0.0506	1.32E-05	1331010
Morningness	rs28362672	A	G	-0.014	0.002	0.7406	3.21E-05	1331010
Morningness	rs2706762	T	C	-0.019	0.003	0.15	3.01E-07	1331010
Morningness	rs7586062	C	G	-0.022	0.002	0.4717	3.06E-21	1331010
Morningness	rs10190053	A	C	-0.013	0.002	0.3695	6.51E-06	1331010
Morningness	rs75863239	T	C	-0.023	0.004	0.9098	3.21E-05	1331010
Morningness	rs62172117	A	G	-0.019	0.002	0.3572	1.43E-14	1331010
Morningness	rs2731553	A	C	-0.016	0.002	0.7544	1.10E-07	1331010
Morningness	rs7579662	A	G	-0.012	0.002	0.3919	2.24E-05	1331010
Morningness	rs13004345	T	C	-0.012	0.002	0.6523	3.76E-05	1331010
Morningness	rs11677484	T	G	0.015	0.002	0.2531	6.25E-07	1331010
Morningness	rs4850712	T	G	0.013	0.002	0.3221	9.40E-06	1331010
Morningness	rs6716898	A	G	0.021	0.002	0.4838	2.32E-19	1331010
Morningness	rs184033703	A	G	0.032	0.005	0.0576	9.65E-07	1331010
Morningness	rs35333999	T	C	-0.047	0.005	0.043	5.10E-16	1331010
Morningness	rs77942338	T	C	-0.056	0.007	0.9737	1.40E-13	1331010
Morningness	rs12477345	T	C	-0.013	0.002	0.6316	1.81E-06	1331010
Morningness	rs11900963	A	T	0.045	0.004	0.9138	3.22E-29	1331010
Morningness	rs62182135	A	C	-0.013	0.002	0.3305	7.80E-06	1331010
Morningness	rs17786957	C	G	-0.018	0.003	0.1633	6,05E-10	1331010
Morningness	rs7428484	A	G	0.013	0.002	0.6734	1.97E-05	1331010
Morningness	rs56411893	A	G	-0.021	0.003	0.8413	1.83E-09	1331010
Morningness	rs62263597	A	G	0.028	0.004	0.0845	4.79E-10	1331010
Morningness	rs11714286	A	G	0.014	0.002	0.5372	9.05E-08	1331010
Morningness	rs7652260	C	G	0.017	0.003	0.8411	1,99E-08	1331010
Morningness	rs67000219	T	C	-0.019	0.003	0.1137	2,68E-08	1331010
Morningness	rs9876864	A	T	0.016	0.002	0.4128	9.20E-11	1331010
Morningness	rs55753638	T	C	-0.022	0.003	0.8786	2.86E-08	1331010
Morningness	rs1800828	C	G	0.014	0.002	0.7473	5.32E-06	1331010
Morningness	rs6799356	A	C	0.014	0.003	0.7538	4,14E-08	1331010
Morningness	rs2699869	A	C	0.013	0.002	0.454	5.06E-06	1331010
Morningness	rs1109088	A	G	0.013	0.002	0.5738	2.60E-06	1331010
Morningness	rs6769642	A	C	0.016	0.002	0.4815	2.80E-10	1331010
Morningness	rs3850174	A	T	-0.014	0.002	0.2541	9.06E-06	1331010
Morningness	rs6443788	A	C	-0.015	0.002	0.2886	1.04E-06	1331010
Morningness	rs6443810	C	G	0.015	0.002	0.6856	2.47E-07	1331010
Morningness	rs6778003	T	G	0.016	0.002	0.2941	4.94E-09	1331010
Morningness	rs7617588	T	C	-0.021	0.003	0.8605	3.49E-08	1331010
Morningness	rs9683585	C	G	-0.012	0.002	0.5608	1.79E-05	1331010

Morningness	rs56040212	A	G	-0.014	0.003	0.2421	2.20E-05	1331010
Morningness	rs28634184	T	C	-0.014	0.002	0.251	2.31E-05	1331010
Morningness	rs57180764	A	G	0.017	0.003	0.7676	3.22E-08	1331010
Morningness	rs4241964	T	G	-0.015	0.002	0.5248	3.54E-09	1331010
Morningness	rs7449161	T	G	0.013	0.002	0.5226	2.20E-06	1331010
Morningness	rs3797051	T	C	0.015	0.003	0.7613	1.48E-06	1331010
Morningness	rs542867945	A	C	0.49	0.089	0.9998	4.29E-05	1331010
Morningness	rs66507804	T	C	-0.017	0.003	0.7946	1.47E-07	1331010
Morningness	rs12657776	A	G	0.019	0.002	0.7482	3.51E-11	1331010
Morningness	rs304137	A	G	0.016	0.002	0.5225	3.06E-11	1331010
Morningness	rs40465	T	G	-0.013	0.002	0.6702	2.46E-05	1331010
Morningness	rs286808	T	C	0.013	0.002	0.4746	3.87E-06	1331010
Morningness	rs10052000	C	G	0.02	0.002	0.3226	7.50E-16	1331010
Morningness	rs2910032	T	C	0.019	0.002	0.5184	6.18E-15	1331010
Morningness	rs42210	C	G	-0.014	0.002	0.7134	3.53E-06	1331010
Morningness	rs335433	T	C	-0.013	0.002	0.4568	2.29E-06	1331010
Morningness	rs9395520	T	C	0.02	0.002	0.3042	1.60E-15	1331010
Morningness	rs139757732	T	C	-0.029	0.005	0.956	2.52E-05	1331010
Morningness	rs9295795	T	C	-0.026	0.005	0.9431	1.90E-05	1331010
Morningness	rs486416	A	G	-0.013	0.002	0.6376	6.07E-06	1331010
Morningness	rs734597	A	G	0.016	0.003	0.1696	1,62E-08	1331010
Morningness	rs11969918	A	G	-0.013	0.002	0.431	1.97E-06	1331010
Morningness	rs144870624	T	C	-0.039	0.007	0.9759	1.92E-05	1331010
Morningness	rs2653349	A	G	0.031	0.003	0.213	1.03E-29	1331010
Morningness	rs9396083	A	G	-0.017	0.002	0.4077	2.45E-12	1331010
Morningness	rs2881955	T	C	0.016	0.002	0.2777	5.64E-08	1331010
Morningness	rs9375352	A	T	0.014	0.002	0.2672	2.03E-05	1331010
Morningness	rs4557564	A	G	0.025	0.004	0.9296	2.15E-06	1331010
Morningness	rs6935086	T	C	0.02	0.003	0.8871	5,87E-09	1331010
Morningness	rs827751	A	G	0.019	0.002	0.3281	2,21E-17	1331010
Morningness	rs9479402	T	C	-0.102	0.01	0.9893	1.35E-19	1331010
Morningness	rs62436127	C	G	0.026	0.003	0.1596	1.43E-15	1331010
Morningness	rs9348050	T	C	0.013	0.002	0.4891	2.00E-06	1331010
Morningness	rs16873715	A	T	-0.015	0.003	0.7564	4.42E-05	1331010
Morningness	rs56382918	T	C	0.018	0.002	0.7436	1.13E-09	1331010
Morningness	rs10236197	T	C	0.014	0.002	0.6303	2.03E-07	1331010
Morningness	rs56049037	A	G	-0.017	0.002	0.2874	2.28E-09	1331010
Morningness	rs4245555	T	C	-0.018	0.002	0.5872	1.96E-12	1331010
Morningness	rs2190500	C	G	-0.022	0.004	0.1025	1.97E-07	1331010
Morningness	rs2138759	A	G	0.014	0.002	0.297	1.75E-06	1331010
Morningness	rs2922966	A	G	0.025	0.003	0.8357	1,11E-17	1331010
Morningness	rs202157	T	C	-0.018	0.002	0.7019	1.21E-11	1331010
Morningness	rs73405966	T	G	0.019	0.003	0.8233	3.61E-06	1331010

Morningness	rs1047998	T	C	0.015	0.002	0.2527	3,39E-09	1331010
Morningness	rs77655131	T	C	-0.022	0.003	0.1223	4.65E-08	1331010
Morningness	rs60984271	A	T	-0.02	0.003	0.8144	2.91E-07	1331010
Morningness	rs112613078	A	G	-0.025	0.003	0.81	1.55E-17	1331010
Morningness	rs17474942	T	C	0.062	0.007	0.0216	4.07E-14	1331010
Morningness	rs148266530	T	C	0.044	0.008	0.0203	1.23E-05	1331010
Morningness	rs79055896	A	G	0.033	0.005	0.9426	4,65E-10	1331010
Morningness	rs6944123	T	G	0.018	0.002	0.3146	1.83E-12	1331010
Morningness	rs10262462	A	G	-0.015	0.002	0.3974	3.18E-09	1331010
Morningness	rs6978514	T	C	-0.013	0.002	0.559	4.87E-05	1331010
Morningness	rs2971970	T	G	0.017	0.003	0.2177	2,23E-11	1331010
Morningness	rs35748596	T	G	-0.016	0.002	0.6423	2.37E-09	1331010
Morningness	rs34344642	T	G	-0.024	0.004	0.0783	2.88E-06	1331010
Morningness	rs12541362	A	T	-0.018	0.002	0.6511	5.55E-12	1331010
Morningness	rs1996866	A	T	-0.016	0.003	0.7498	2.31E-05	1331010
Morningness	rs1919346	A	G	0.012	0.002	0.5251	1.30E-05	1331010
Morningness	rs11988076	A	G	-0.019	0.003	0.8359	1.82E-08	1331010
Morningness	rs6472936	T	C	0.018	0.003	0.7788	5.16E-09	1331010
Morningness	rs1110275	T	C	0.02	0.004	0.9058	4.05E-05	1331010
Morningness	rs34578339	A	T	-0.016	0.003	0.843	2.84E-05	1331010
Morningness	rs17748165	A	G	0.014	0.002	0.7422	3.05E-05	1331010
Morningness	rs72673588	C	G	-0.017	0.003	0.8152	1.26E-06	1331010
Morningness	rs3100052	A	G	0.013	0.002	0.3874	1.30E-05	1331010
Morningness	rs2737245	T	G	0.018	0.002	0.2779	2.94E-11	1331010
Morningness	rs10758971	T	C	0.012	0.002	0.652	4,58E-08	1331010
Morningness	rs1323591	T	C	-0.016	0.002	0.3243	2.11E-09	1331010
Morningness	rs1936637	T	C	0.013	0.002	0.6601	9.25E-06	1331010
Morningness	rs10973160	T	C	-0.013	0.002	0.3195	1.71E-05	1331010
Morningness	rs2291589	T	G	0.018	0.002	0.6226	1.65E-13	1331010
Morningness	rs78146603	T	C	0.033	0.006	0.9618	2,93E-09	1331010
Morningness	rs77598468	A	C	-0.042	0.006	0.0343	1.05E-09	1331010
Morningness	rs72742394	T	C	0.021	0.004	0.9017	7.04E-06	1331010
Morningness	rs4565536	A	C	-0.012	0.002	0.4993	4.97E-05	1331010
Morningness	rs10797119	T	C	-0.013	0.002	0.4633	2.80E-06	1331010
Morningness	rs10818834	T	C	0.014	0.002	0.7344	5.24E-06	1331010
Morningness	rs28365587	A	G	-0.013	0.002	0.5141	3.60E-06	1331010
Morningness	rs10448340	T	G	-0.013	0.002	0.6806	9.07E-06	1331010
Morningness	rs28458909	T	C	-0.03	0.004	0.1224	1.95E-13	1331010
Morningness	rs1750785	A	G	0.014	0.002	0.2638	3.52E-06	1331010
Morningness	rs9416744	A	C	0.017	0.002	0.2561	1.47E-09	1331010
Morningness	rs7910164	A	G	0.012	0.002	0.6423	4,83E-08	1331010
Morningness	rs76518095	T	C	0.023	0.004	0.0751	1.00E-05	1331010
Morningness	rs9795439	A	G	0.018	0.003	0.1963	6,68E-09	1331010

Morningness	rs925947	T	G	0.016	0.003	0.2016	9.64E-07	1331010
Morningness	rs605765	T	C	-0.012	0.002	0.6107	3.13E-05	1331010
Morningness	rs12799529	T	C	0.018	0.003	0.7583	5.13E-10	1331010
Morningness	rs11032362	A	G	0.031	0.004	0.0911	3.46E-14	1331010
Morningness	rs34239319	T	G	0.024	0.004	0.1018	6.45E-09	1331010
Morningness	rs4319473	A	G	0.017	0.003	0.1573	9.45E-06	1331010
Morningness	rs6485751	C	G	-0.016	0.003	0.2097	1.44E-06	1331010
Morningness	rs11039308	A	G	0.014	0.002	0.4083	4,94E-11	1331010
Morningness	rs11605681	A	G	0.027	0.005	0.0563	1.21E-05	1331010
Morningness	rs3168135	A	G	-0.017	0.003	0.2408	3.64E-09	1331010
Morningness	rs4008953	A	G	-0.016	0.002	0.7123	3.23E-08	1331010
Morningness	rs4237555	T	C	0.012	0.002	0.5272	4.19E-05	1331010
Morningness	rs4936290	A	C	-0.013	0.002	0.6508	1.18E-05	1331010
Morningness	rs577924	T	C	0.012	0.002	0.5342	2.07E-05	1331010
Morningness	rs1174510	A	G	0.012	0.002	0.6244	4.86E-05	1331010
Morningness	rs11611435	T	C	0.012	0.002	0.5528	4.00E-05	1331010
Morningness	rs13377754	T	C	0.024	0.002	0.6123	7.84E-24	1331010
Morningness	rs11610973	T	C	-0.024	0.004	0.8737	3.08E-08	1331010
Morningness	rs7313852	A	G	-0.025	0.002	0.435	1.02E-26	1331010
Morningness	rs117554039	A	G	0.021	0.004	0.9121	4.59E-05	1331010
Morningness	rs12828063	T	G	-0.014	0.002	0.691	4.93E-06	1331010
Morningness	rs11183201	T	C	-0.017	0.002	0.4925	1.60E-11	1331010
Morningness	rs671255	A	G	-0.014	0.002	0.7505	1.42E-05	1331010
Morningness	rs7299922	A	G	0.013	0.002	0.6334	8.56E-06	1331010
Morningness	rs9788226	C	G	0.022	0.003	0.8781	1.25E-08	1331010
Morningness	rs7138306	A	G	0.013	0.002	0.4693	8,86E-10	1331010
Morningness	rs7488974	A	G	0.016	0.002	0.3976	1.81E-10	1331010
Morningness	rs10861694	T	C	0.014	0.002	0.5641	2.60E-07	1331010
Morningness	rs77531286	T	C	-0.017	0.003	0.8517	1.40E-05	1331010
Morningness	rs4102203	T	C	0.024	0.003	0.8936	7.80E-09	1331010
Morningness	rs61963123	T	C	0.017	0.003	0.7847	2.33E-07	1331010
Morningness	rs9597250	A	C	-0.019	0.003	0.188	2.88E-09	1331010
Morningness	rs2321993	A	G	-0.014	0.002	0.5691	2.59E-08	1331010
Morningness	rs2593487	A	G	-0.014	0.002	0.3388	2.88E-07	1331010
Morningness	rs9565309	T	C	0.066	0.006	0.9662	1.26E-25	1331010
Morningness	rs61963491	A	G	0.112	0.016	0.9942	1.60E-09	1331010
Morningness	rs7337911	A	G	0.015	0.002	0.7471	1.00E-06	1331010
Morningness	rs9521184	T	C	0.013	0.002	0.4871	8.34E-07	1331010
Morningness	rs11157143	A	G	-0.015	0.002	0.7264	8.23E-07	1331010
Morningness	rs56376592	A	C	0.017	0.003	0.8217	1.40E-06	1331010
Morningness	rs4899502	A	G	-0.016	0.002	0.6869	1.61E-08	1331010
Morningness	rs12432176	A	C	0.013	0.002	0.3781	1,47E-08	1331010
Morningness	rs2701524	T	C	0.013	0.002	0.5871	5.39E-06	1331010

Morningness	rs59986227	C	G	-0.015	0.002	0.7456	5.36E-06	1331010
Morningness	rs12442008	T	C	0.014	0.002	0.2521	3.36E-05	1331010
Morningness	rs11852820	C	G	-0.014	0.002	0.6589	5.76E-07	1331010
Morningness	rs2304467	C	G	-0.012	0.002	0.6026	3,91E-08	1331010
Morningness	rs7196720	T	C	0.013	0.002	0.486	4.32E-06	1331010
Morningness	rs12927162	A	G	0.028	0.002	0.7237	2.11E-29	1331010
Morningness	rs1421085	T	C	-0.023	0.002	0.5961	1.39E-22	1331010
Morningness	rs1110698	A	C	-0.022	0.004	0.0788	2.89E-05	1331010
Morningness	rs2398144	A	C	-0.021	0.002	0.3947	2.42E-18	1331010
Morningness	rs8044054	T	C	0.015	0.002	0.3889	3.87E-09	1331010
Morningness	rs11641811	A	C	-0.013	0.002	0.5218	5.77E-07	1331010
Morningness	rs17604349	A	G	-0.022	0.003	0.1792	1.21E-12	1331010
Morningness	rs58356259	T	C	-0.013	0.002	0.4687	2.38E-06	1331010
Morningness	rs2518022	T	C	0.032	0.004	0.0856	1.53E-13	1331010
Morningness	rs1242490	C	G	-0.018	0.002	0.7456	7,33E-14	1331010
Morningness	rs2232839	T	C	-0.025	0.003	0.2113	2.41E-18	1331010
Morningness	rs9915731	A	T	-0.013	0.002	0.2703	2.80E-05	1331010
Morningness	rs225289	T	C	0.016	0.003	0.1693	1.24E-05	1331010
Morningness	rs72828815	T	C	0.016	0.003	0.1849	6.08E-06	1331010
Morningness	rs117974417	C	G	-0.018	0.003	0.8477	1.54E-06	1331010
Morningness	rs6504758	A	G	-0.014	0.002	0.4645	9.83E-08	1331010
Morningness	rs9898091	T	C	0.038	0.006	0.9604	6.88E-09	1331010
Morningness	rs8072058	A	T	-0.015	0.003	0.7822	1.38E-05	1331010
Morningness	rs17682747	A	G	0.014	0.003	0.2312	4.69E-05	1331010
Morningness	rs2916142	T	C	0.015	0.002	0.4565	7.63E-09	1331010
Morningness	rs10491171	C	G	0.019	0.003	0.865	3.62E-06	1331010
Morningness	rs487952	A	G	0.013	0.002	0.5656	4.43E-06	1331010
Morningness	rs974552	A	G	-0.022	0.003	0.8085	1.40E-12	1331010
Morningness	rs1013987	T	C	-0.015	0.002	0.4042	1.36E-08	1331010
Morningness	rs4239386	A	T	-0.019	0.002	0.3342	2,06E-17	1331010
Morningness	rs12969848	T	C	0.017	0.002	0.5294	1.52E-12	1331010
Morningness	rs989885	A	G	-0.024	0.004	0.9368	3.83E-05	1331010
Morningness	rs9956387	A	T	-0.014	0.002	0.4963	1.38E-07	1331010
Morningness	rs17596722	T	C	-0.018	0.003	0.2006	3.24E-08	1331010
Morningness	rs66791238	T	C	-0.015	0.003	0.7917	2.93E-05	1331010
Morningness	rs12959908	T	C	-0.012	0.002	0.5776	1.10E-05	1331010
Morningness	rs8094095	A	G	0.018	0.003	0.1496	5.92E-06	1331010
Morningness	rs9964420	A	C	-0.022	0.002	0.301	4.03E-18	1331010
Morningness	rs11152350	A	C	-0.015	0.002	0.4704	1.07E-09	1331010
Morningness	rs9958145	A	G	-0.015	0.003	0.2009	4.26E-05	1331010
Morningness	rs10402849	T	C	0.016	0.003	0.2008	6.73E-06	1331010
Morningness	rs3843751	T	C	0.013	0.002	0.6642	6.33E-06	1331010
Morningness	rs9636202	A	G	-0.013	0.002	0.2653	3.53E-05	1331010

Morningness	rs12481462	T	C	0.012	0.002	0.3727	4.36E-05	1331010
Morningness	rs78095690	T	C	-0.013	0.002	0.5635	5.75E-06	1331010
Morningness	rs6131942	A	G	-0.015	0.002	0.4196	7.63E-09	1331010
Morningness	rs1737893	T	C	-0.014	0.002	0.3779	1.37E-06	1331010
Morningness	rs2072727	T	C	0.012	0.002	0.4359	3.46E-05	1331010
Morningness	rs695459	T	C	-0.013	0.002	0.3886	6.59E-06	1331010
Morningness	rs28580373	A	G	0.014	0.003	0.2317	3.25E-05	1331010
Morningness	rs11705370	A	T	-0.019	0.002	0.5981	3.43E-12	1331010
Morningness	rs9611302	A	G	-0.019	0.003	0.1142	8.37E-06	1331010
Morningness	rs55715427	C	G	-0.022	0.004	0.097	1,35E-09	1331010
Morningness	rs4822107	A	G	0.012	0.002	0.5065	2.23E-05	1331010
Morningness	rs2294203	A	G	0.013	0.002	0.6796	2.97E-05	1331010
Morningness	rs196973	A	C	-0.015	0.002	0.5395	5.95E-06	1331010
Morningness	rs12844850	A	G	-0.013	0.002	0.3614	3.71E-17	1331010
Morningness	rs213443	C	G	0.013	0.002	0.4522	6,36E-10	1331010
Morningness	rs4375148	T	C	0.015	0.003	0.769	2.18E-09	1331010
Morningness	rs6621219	T	C	0.026	0.003	0.1754	1.07E-07	1331010
Morningness	rs5987510	A	G	0.014	0.002	0.3743	2.09E-06	1331010

Table S8. The summary information for instrumental variables of snoring.

phenotype	SNP	Effect allele	Other allele	beta	se	eaf	P val	Sample size
Snoring	rs35915391	C	G	0.97	0.005214	0.3513	2.25E-08	1331010
Snoring	rs35562935	A	G	1.06	0.009454	0.0746	1.69E-08	1331010
Snoring	rs10190879	T	C	0.97	0.005481	0.298	1.81E-08	1331010
Snoring	rs72906130	G	C	1.06	0.007283	0.1345	3.50E-14	1331010
Snoring	rs9309771	A	G	1.03	0.005018	0.4521	5.65E-09	1331010
Snoring	rs34811474	A	G	0.96	0.005929	0.2308	4.22E-11	1331010
Snoring	rs6855873	T	C	1.03	0.005822	0.2411	2.08E-08	1331010
Snoring	rs2307111	C	T	0.97	0.005108	0.3946	1.34E-11	1331010
Snoring	rs10062026	A	G	0.97	0.005204	0.3591	2.08E-08	1331010
Snoring	rs745558	A	G	0.97	0.005347	0.3279	2.66E-10	1331010
Snoring	rs947612	G	A	1.03	0.005761	0.2474	9.92E-09	1331010
Snoring	rs17060460	G	A	1.03	0.005955	0.2256	1.43E-08	1331010
Snoring	rs9389081	A	T	0.95	0.00946	0.0764	4.37E-08	1331010
Snoring	rs2981329	C	T	1.03	0.00579	0.2551	7.74E-09	1331010
Snoring	rs7007887	T	C	1.04	0.005024	0.4339	9.88E-14	1331010
Snoring	rs4523230	A	T	0.97	0.005572	0.2832	1.67E-09	1331010
Snoring	rs1016013	A	G	0.97	0.005145	0.4036	7.22E-10	1331010
Snoring	rs11256034	T	C	1.04	0.005772	0.2478	4.67E-11	1331010
Snoring	rs2049045	C	G	0.96	0.006417	0.1866	8.87E-11	1331010
Snoring	rs10878269	T	C	1.04	0.005176	0.3647	3.76E-13	1331010
Snoring	rs12427782	T	G	0.97	0.005055	0.491	5.77E-09	1331010
Snoring	rs2762049	C	G	1.04	0.005107	0.3914	8.69E-12	1331010
Snoring	rs2408111	A	T	1.05	0.007367	0.1329	3.00E-09	1331010
Snoring	rs592333	G	A	1.04	0.005012	0.4433	1.08E-16	1331010
Snoring	rs2664299	C	T	0.97	0.005067	0.4187	4.48E-11	1331010
Snoring	rs9933881	C	T	1.06	0.00948	0.0743	8.94E-09	1331010
Snoring	rs732172	T	C	1.03	0.005154	0.3709	1.35E-09	1331010
Snoring	rs8047587	T	G	1.03	0.005036	0.4396	2.13E-09	1331010
Snoring	rs7217107	G	A	0.97	0.005429	0.3111	3.52E-09	1331010
Snoring	rs12449843	A	G	0.97	0.005151	0.378	4.45E-10	1331010
Snoring	rs1641511	G	A	1.03	0.005956	0.2355	1.40E-08	1331010
Snoring	rs57222984	G	A	1.04	0.005824	0.2423	3.03E-11	1331010
Snoring	rs4792897	G	A	0.96	0.006603	0.1738	2.74E-08	1331010
Snoring	rs1563304	T	C	1.04	0.006506	0.1782	3.72E-08	1331010
Snoring	rs2924251	A	G	1.03	0.005307	0.3325	5.17E-09	1331010
Snoring	rs79275904	T	C	0.95	0.008855	0.0882	5.64E-09	1331010
Snoring	rs180107	A	T	0.97	0.00507	0.4093	3.86E-09	1331010
Snoring	rs4987719	T	C	1.08	0.01368	0.0338	3.59E-08	1331010
Snoring	rs10415992	G	C	0.95	0.008565	0.0947	8.14E-09	1331010
Snoring	rs34107769	C	T	0.97	0.005485	0.2932	9.93E-09	1331010
Snoring	rs6099273	T	C	1.03	0.005762	0.2496	1.22E-08	1331010

Snoring	rs17435	T	A	0.96	0.007573	0.181	3.80E-08	1331010

eaf: effect allele frequency; SE: standard error.

TableS9. SNPs used as instrumental variables from sleep traits and RA GWASs (P < 5× 10^{-8}).

Exposure	SNP	Effect allele	Other allele	Exposure			Outcome			Proxy SNP	Target effect allele	Target other allele
				Beta	SE	*P* value	Beta	SE	*P* value			
Short sleep	rs11763750	G	A	0.035367144	0.006052001	5.10E-09	0.0304592	0.021678	0.16	-	-	-
	rs1229762	T	C	0.037295785	0.005240102	1.10E-12	0.0392207	0.0197456	0.0470002	-	-	-
	rs12518468	C	T	0.031498667	0.005470199	8.50E-09	0.0100503	0.0164003	0.54	-	-	-
	rs12567114	G	A	0.036331929	0.006178787	4.10E-09	-0.0198026	0.0293594	0.5	-	-	-
	rs12661667	T	C	0.027615167	0.004972567	2.80E-08	0.0198026	0.0220895	0.37	-	-	-
	rs12963463	C	T	0.028587457	0.004258199	1.90E-11	0.0100503	0.0221489	0.649999	-	-	-
	rs13107325	T	C	0.075107472	0.010262117	2.50E-13	-0.0304592	0.0431249	0.48	-	-	-
	rs1380703	G	A	0.035367144	0.005248498	1.60E-11	0.040822	0.0237009	0.0850002	-	-	-
	rs1607227	G	T	0.030529205	0.005050854	1.50E-09	0.0100503	0.0377392	0.79	-	-	-
	rs17005118	A	G	0.030529205	0.005121099	2.50E-09	0.0392207	0.025226	0.12	-	-	-
	rs17388803	C	A	0.05259245	0.008513132	6.50E-10	0.0100503	0.0377392	0.79	-	-	-
	rs2014830	C	T	0.029558802	0.005316471	2.70E-08	-0.0198026	0.0175806	0.26	-	-	-
	rs205024	C	T	0.030529205	0.005491008	2.70E-08	0.0100503	0.0133044	0.450001	-	-	-
	rs2820313	G	A	0.030529205	0.005109436	2.30E-09	0.0202027	0.0273436	0.46	-	-	-
	rs2863957	C	A	0.054488185	0.006243162	2.60E-18	0.0202027	0.0211732	0.34	-	-	-
	rs3776864	A	C	0.031498667	0.00558482	1.70E-08	0.0582689	0.0185677	0.0017	-	-	-
	rs4585442	G	A	0.030529205	0.004969791	8.10E-10	0.0100503	0.0191654	0.6	-	-	-
	rs5757675	G	T	0.034401427	0.005782851	2.70E-09	0.0202027	0.017194	0.24	-	-	-
	rs59779556	T	G	0.024692613	0.004399966	2.00E-08	0.00995033	0.0151029	0.51	-	-	-
	rs7524118	C	T	0.029558802	0.00541876	4.90E-08	-0.00995033	0.0189747	0.6	-	-	-
	rs7939345	T	G	0.035367144	0.006441103	4.00E-08	-0.040822	0.024302	0.0929994	-	-	-
	rs9321171	C	T	0.031498667	0.005745595	4.20E-08	0.0202027	0.0220707	0.36	-	-	-

frequent insomnia	rs10156602	A	G	0.039220713	0.005635068	3.40E-12	0.00995033	0.0277586	0.719999	-	-	-
	rs10838708	G	A	0.029558802	0.004958319	2.50E-09	-0.0198026	0.0203289	0.33	-	-	-
	rs11097861	G	A	0.039220713	0.00651058	1.70E-09	-0.00995033	0.0147524	0.5	-	-	-
	rs113851554	T	G	0.182321557	0.013491706	1.30E-41	0.00995033	0.0437291	0.82	rs11679120	T	G
	rs11635495	C	T	0.039220713	0.006767103	6.80E-09	-0.0198026	0.0164973	0.23	-	-	-
	rs11673344	G	A	0.039220713	0.006402123	9.00E-10	0.0202027	0.0194925	0.3	-	-	-
	rs11793074	A	G	0.039220713	0.007581378	2.30E-07	-0.0512933	0.0276935	0.064	-	-	-
	rs11804386	A	G	0.039220713	0.007045927	2.60E-08	-0.0100503	0.0359816	0.780001	-	-	-
	rs1430205	T	C	0.029558802	0.00536513	3.60E-08	0.0198026	0.0250923	0.43	-	-	-
	rs17139246	C	T	0.029558802	0.005387562	4.10E-08	0.0304592	0.0211591	0.15	-	-	-
	rs17151854	T	G	0.058268908	0.010441754	2.40E-08	-0.00995033	0.0392755	0.8	rs10087341	T	G
	rs17669584	G	A	0.039220713	0.007118835	3.60E-08	0.0100503	0.0235841	0.67	-	-	-
	rs1841625	G	A	0.029558802	0.00536513	3.60E-08	0.0100503	0.0186519	0.59	-	-	-
	rs1942262	A	G	0.039220713	0.005279902	1.10E-13	0.0295588	0.0251567	0.24	-	-	-
	rs2062113	T	C	0.039220713	0.006157858	1.90E-10	-0.0202027	0.0161162	0.21	-	-	-
	rs2296580	G	T	0.048790164	0.007147152	8.70E-12	0.0100503	0.0124629	0.42	-	-	-
	rs2613503	A	C	0.039220713	0.007635786	2.80E-07	0.0392207	0.0279137	0.16	-	-	-
	rs2956278	G	A	0.048790164	0.008580656	1.30E-08	-0.00995033	0.0325727	0.760001	rs4882414	G	A
	rs302165	G	A	0.039220713	0.008315735	2.40E-06	0.0202027	0.0198998	0.31	-	-	-
	rs314280	G	A	0.039220713	0.006250475	3.50E-10	-0.0198026	0.0161453	0.22	-	-	-
	rs324017	A	C	0.048790164	0.007561404	1.10E-10	0.0198026	0.028037	0.48	-	-	-
	rs3824081	T	C	0.039220713	0.006863317	1.10E-08	-0.0100503	0.0164003	0.54	-	-	-
	rs4577309	A	G	0.029558802	0.005012451	3.70E-09	-0.0304592	0.020117	0.13	-	-	-
	rs4688760	T	C	0.048790164	0.006842454	1.00E-12	-0.0100503	0.0124629	0.42	-	-	-
	rs4751	T	G	0.029558802	0.005231202	1.60E-08	0.0676586	0.0386469	0.08	-	-	-

	rs62158170	A	G	0.048790164	0.006769386	5.70E-13	0.0295588	0.0220464	0.18	-	-	-
	rs6593005	G	A	0.029558802	0.005135074	8.60E-09	-0.00995033	0.0158443	0.53	-	-	-
	rs6664467	G	A	0.048790164	0.008919568	4.50E-08	-0.00995033	0.0175166	0.57	-	-	-
	rs6785034	A	G	0.029558802	0.007578237	9.60E-05	-0.0100503	0.0302855	0.74	rs7638072	A	G
	rs68094047	T	C	0.039220713	0.006629675	3.30E-09	-0.0100503	0.0280376	0.719999	rs35393419	T	C
	rs7711696	T	G	0.048790164	0.007166647	9.90E-12	0.00995033	0.0233495	0.67	-	-	-
	rs9845387	C	A	0.09531018	0.017008879	2.10E-08	0.0202027	0.0524309	0.7	-	-	-
any insomnia	rs10156602	A	G	0.029558802	0.005186004	1.20E-08	0.00995033	0.0277586	0.719999	-	-	-
	rs10838708	G	A	0.019802627	0.004558762	1.40E-05	-0.0198026	0.0203289	0.33	-	-	-
	rs11097861	G	A	0.029558802	0.005023425	4.00E-09	-0.00995033	0.0147524	0.5	-	-	-
	rs113851554	T	G	0.131028262	0.011853626	2.10E-28	0.00995033	0.0437291	0.82	rs11679120	T	G
	rs11635495	C	T	0.019802627	0.004274041	3.60E-06	-0.0198026	0.0164973	0.23	-	-	-
	rs11673344	G	A	0.029558802	0.00548541	7.10E-08	0.0202027	0.0194925	0.3	-	-	-
	rs11793074	A	G	0.039220713	0.006799167	8.00E-09	-0.0512933	0.0276935	0.064	-	-	-
	rs11804386	A	G	0.029558802	0.005104332	7.00E-09	-0.0100503	0.0359816	0.780001	-	-	-
	rs1430205	T	C	0.029558802	0.005497905	7.60E-08	0.0198026	0.0250923	0.43	-	-	-
	rs17139246	C	T	0.019802627	0.004541778	1.30E-05	0.0304592	0.0211591	0.15	-	-	-
	rs17151854	T	G	0.029558802	0.007493865	8.00E-05	-0.00995033	0.0392755	0.8	rs10087341	T	G
	rs17669584	G	A	0.039220713	0.007194731	5.00E-08	0.0100503	0.0235841	0.67	-	-	-
	rs1841625	G	A	0.019802627	0.004483099	1.00E-05	0.0100503	0.0186519	0.59	-	-	-
	rs1942262	A	G	0.029558802	0.00445046	3.10E-11	0.0295588	0.0251567	0.24	-	-	-
	rs2296580	G	T	0.039220713	0.007009344	2.20E-08	0.0100503	0.0124629	0.42	-	-	-
	rs2613503	A	C	0.029558802	0.00541876	4.90E-08	0.0392207	0.0279137	0.16	-	-	-
	rs28061	A	G	0.029558802	0.005796063	3.40E-07	0.0676586	0.0175914	0.00012	-	-	-
	rs2956278	G	A	0.029558802	0.005731003	2.50E-07	-0.00995033	0.0325727	0.760001	rs4882414	G	A

	rs302165	G	A	0.029558802	0.005350275	3.30E-08	0.0202027	0.0198998	0.31	-	-	-
	rs3104778	A	G	0.019802627	0.003839429	2.50E-07	0.0198026	0.0147698	0.18	-	-	-
	rs324017	A	C	0.029558802	0.005282612	2.20E-08	0.0198026	0.028037	0.48	-	-	-
	rs3824081	T	C	0.039220713	0.006198959	2.50E-10	-0.0100503	0.0164003	0.54	-	-	-
	rs4577309	A	G	0.029558802	0.005275009	2.10E-08	-0.0304592	0.020117	0.13	-	-	-
	rs4688760	T	C	0.039220713	0.005724268	7.30E-12	-0.0100503	0.0124629	0.42	-	-	-
	rs4751	T	G	0.019802627	0.00494907	6.30E-05	0.0676586	0.0386469	0.08	-	-	-
	rs62158170	A	G	0.048790164	0.005897107	1.30E-16	0.0295588	0.0220464	0.18	-	-	-
	rs6593005	G	A	0.019802627	0.00409166	1.30E-06	-0.00995033	0.0158443	0.53	-	-	-
	rs6664467	G	A	0.029558802	0.006603617	7.60E-06	-0.00995033	0.0175166	0.57	-	-	-
	rs6785034	A	G	0.029558802	0.005355358	3.40E-08	-0.0100503	0.0302855	0.74	rs7638072	A	G
	rs68094047	T	C	0.029558802	0.005704579	2.20E-07	-0.0100503	0.0280376	0.719999	rs35393419	T	C
	rs6932158	C	T	0.029558802	0.006779378	1.30E-05	-0.0392207	0.0179624	0.0290001	-	-	-
	rs7711696	T	G	0.029558802	0.005776291	3.10E-07	0.00995033	0.0233495	0.67	-	-	-
	rs9845387	C	A	0.067658648	0.01383149	1.00E-06	0.0202027	0.0524309	0.7	-	-	-
sleep duration	rs10973207	T	G	0.02	0.003391	2.02E-06	0.0100503	0.0359816	0.780001	rs10814514	T	G
	rs11076146	G	T	0.01	0.002461	2.96E-06	-0.00995033	0.0162371	0.54	-	-	-
	rs11621908	T	C	-0.03	0.004548	4.95E-06	0.0392207	0.040263	0.33	-	-	-
	rs11650677	A	G	0.01	0.00258	8.95E-09	-0.0100503	0.0235841	0.67	-	-	-
	rs12215241	A	G	-0.02	0.002931	6.09E-10	0.0861777	0.0241543	0.000359998	-	-	-
	rs12607679	C	T	-0.02	0.002821	9.79E-08	0.0202027	0.0164715	0.22	-	-	-
	rs1263056	G	A	-0.01	0.002471	4.15E-05	0.0100503	0.0168133	0.55	-	-	-
	rs13109404	G	T	-0.03	0.004795	1.13E-08	-0.076961	0.0382113	0.0439997	-	-	-
	rs13329140	A	G	-0.02	0.002888	7.51E-08	-0.0202027	0.0273436	0.46	-	-	-
	rs144625846	G	A	-0.02	0.003575	1.05E-05	-0.0202027	0.024521	0.41	rs11191275	G	A

	rs1553132	G	A	0.02	0.002788	6.63E-06	-0.0295588	0.0240996	0.22	-	-	-
	rs16834426	A	G	0.02	0.002763	1.76E-05	-0.0100503	0.015622	0.52	-	-	-
	rs2192528	A	G	0.02	0.002452	6.27E-07	0.0198026	0.0250923	0.43	-	-	-
	rs28651105	G	A	0.02	0.002969	4.75E-05	0.0100503	0.0243666	0.68	-	-	-
	rs365663	G	A	-0.02	0.002462	1.12E-08	0.0100503	0.0302855	0.74	-	-	-
	rs3823624	C	T	0.02	0.003188	1.30E-06	-0.0392207	0.0259036	0.13	-	-	-
	rs41501452	G	A	-0.02	0.002633	4.04E-06	0.0304592	0.021678	0.16	-	-	-
	rs4688116	G	T	-0.01	0.002508	4.20E-05	-0.0198026	0.0164973	0.23	-	-	-
	rs4767550	G	A	0.01	0.002507	2.70E-06	0.0100503	0.0107537	0.35	-	-	-
	rs540431	G	A	-0.01	0.002465	4.91E-06	-0.00995033	0.0258235	0.7	rs269045	G	A
	rs62158206	C	T	0.04	0.002968	3.00E-40	-0.0198026	0.0203289	0.33	-	-	-
	rs651902	C	T	0.01	0.002534	3.35E-09	-0.00995033	0.0212751	0.64	-	-	-
	rs6889592	A	G	0.02	0.002596	1.44E-08	-0.0512933	0.016157	0.0015	-	-	-
	rs7115856	C	A	0.01	0.002452	1.25E-06	0.040822	0.0198768	0.04	-	-	-
	rs7686205	G	A	0.01	0.002519	1.37E-05	-0.0100503	0.0359816	0.780001	rs62304946	G	A
	rs7915425	T	C	0.02	0.003221	3.08E-07	0.0198026	0.0245562	0.42	-	-	-
	rs8047587	T	G	-0.02	0.002468	1.09E-09	-0.0100503	0.0243666	0.68	rs11642015	T	G
	rs9362971	C	T	-0.01	0.002567	1.41E-05	0.0100503	0.0228462	0.66	-	-	-
	rs9451146	T	C	-0.02	0.002915	8.53E-06	0.00995033	0.0437291	0.82	rs4706335	T	C
	rs9843801	C	T	0.02	0.002584	5.82E-10	0.0100503	0.025198	0.69	-	-	-
getting up	rs10175975	T	C	0.01	0.0023	3.30E-06	-0.0304592	0.0293885	0.3	-	-	-
	rs10470887	G	A	-0.01	0.0017	4.05E-05	-0.0198026	0.0175806	0.26	-	-	-
	rs11229264	A	G	-0.01	0.0019	1.43E-07	-0.040822	0.021186	0.0539995	-	-	-
	rs113240734	A	G	0.02	0.0023	2.17E-13	-0.0198026	0.019913	0.32	rs7547493	A	G
	rs11642015	T	C	0.01	0.0018	6.20E-08	-0.0100503	0.0243666	0.68	-	-	-

	rs11643192	A	C	-0.01	0.0018	1.30E-06	-0.0100503	0.0181616	0.58	-	-	-
	rs11697690	C	T	0.01	0.0017	2.33E-05	-0.0198026	0.0179524	0.27	-	-	-
	rs12150229	G	A	0.01	0.0021	3.52E-06	-0.00995033	0.0179809	0.58	-	-	-
	rs12515274	A	G	-0.01	0.002	5.37E-07	0.0198026	0.0274093	0.47	-	-	-
	rs12601968	T	G	-0.01	0.0018	1.16E-06	0.00995033	0.016646	0.55	-	-	-
	rs12736689	C	T	0.05	0.0051	1.66E-21	0.0202027	0.0396074	0.61	-	-	-
	rs12752290	C	T	0.01	0.0017	1.02E-09	-0.0295588	0.0184951	0.11	-	-	-
	rs13116306	T	C	-0.01	0.0018	1.76E-05	0.0676586	0.0210383	0.00129999	-	-	-
	rs1420607	A	G	0.01	0.0019	1.57E-08	0.00995033	0.0241242	0.68	-	-	-
	rs1459192	T	C	-0.01	0.0018	2.50E-05	0.0198026	0.0225569	0.38	-	-	-
	rs1606803	T	C	0.01	0.0019	3.22E-07	-0.0100503	0.0172437	0.56	-	-	-
	rs16917522	C	T	0.01	0.0022	1.12E-06	-0.0295588	0.0291156	0.31	-	-	-
	rs17822102	G	A	0.01	0.0018	9.40E-07	0.0100503	0.0145592	0.49	-	-	-
	rs2053457	C	T	-0.02	0.002	1.29E-12	0.0100503	0.0441686	0.82	-	-	-
	rs2193749	T	C	-0.01	0.0017	1.84E-05	-0.0512933	0.0185539	0.00569994	-	-	-
	rs2653349	A	G	0.02	0.0021	8.45E-26	0.00995033	0.0170721	0.56	-	-	-
	rs2944822	T	C	0.01	0.0017	4.43E-06	-0.0100503	0.0291208	0.73	rs2968500	T	C
	rs301806	C	T	-0.01	0.0017	4.73E-05	-0.0295588	0.0176506	0.0940005	-	-	-
	rs3735478	T	G	-0.01	0.0019	1.57E-05	-0.0202027	0.0194925	0.3	-	-	-
	rs3760185	T	C	-0.01	0.002	4.52E-05	-0.0100503	0.0396702	0.8	-	-	-
	rs406952	C	T	0.01	0.0018	1.20E-05	-0.00995033	0.0162371	0.54	-	-	-
	rs4671328	T	G	0.01	0.0018	1.06E-06	-0.0100503	0.0145592	0.49	-	-	-
	rs4790352	G	A	-0.02	0.0031	8.78E-06	0.0202027	0.0459243	0.66	-	-	-
	rs4853283	G	A	-0.01	0.0017	1.84E-13	0.00995033	0.0356235	0.780001	rs11896560	G	A
	rs4884166	A	G	-0.01	0.0022	1.15E-05	-0.0202027	0.0207396	0.33	-	-	-

	rs4958316	A	C	0.02	0.0019	3.69E-12	-0.0100503	0.0228462	0.66	-	-	-
	rs4962716	T	C	-0.02	0.0026	1.82E-06	-0.0100503	0.0302855	0.74	-	-	-
	rs553108	A	G	0.01	0.0018	3.12E-05	-0.301105	0.0167993	7.70E-72	-	-	-
	rs61773374	G	A	0.02	0.0022	3.37E-14	0.0512933	0.0180559	0.00449997	-	-	-
	rs6575012	A	G	-0.01	0.0017	2.57E-05	-0.0304592	0.0190585	0.11	-	-	-
	rs6581138	A	G	0.01	0.002	2.30E-07	-0.040822	0.0180858	0.0239999	-	-	-
	rs7297799	T	C	-0.01	0.0018	1.21E-09	0.00995033	0.0175166	0.57	-	-	-
	rs7332608	G	A	-0.03	0.0048	4.55E-08	-0.0487902	0.0555762	0.38	-	-	-
	rs76048411	T	C	0.01	0.0017	8.53E-07	0.0198026	0.0256447	0.44	rs72631529	T	C
	rs77576965	T	C	0.01	0.0019	1.94E-05	0.0202027	0.0203153	0.32	rs16851819	T	C
	rs8182491	T	C	-0.02	0.0029	1.18E-06	0.0676586	0.0337508	0.0449997	-	-	-
	rs9399613	T	C	-0.01	0.0019	1.32E-05	-0.0100503	0.0396702	0.8	rs9390489	T	C
	rs9995419	A	G	0.01	0.002	1.50E-05	-0.0100503	0.0168133	0.55	-	-	-
mornings	rs10190053	A	C	-0.013	0.002	6.51E-06	0.0392207	0.0392207	0.11	-	-	-
	rs10262462	A	G	-0.015	0.002	3.18E-09	-0.0304592	-0.0304592	0.1	-	-	-
	rs10758971	T	C	0.012	0.002	9.87E-10	-0.0304592	-0.0304592	0.13	-	-	-
	rs10797119	T	C	-0.013	0.002	2.80E-06	-0.0202027	-0.0202027	0.35	-	-	-
	rs10818834	T	C	0.014	0.002	5.24E-06	-0.0100503	-0.0100503	0.64	-	-	-
	rs10861694	T	C	0.014	0.002	2.60E-07	0.0295588	0.0295588	0.19	-	-	-
	rs11032362	A	G	0.031	0.004	3.46E-14	-0.0202027	-0.0202027	0.37	-	-	-
	rs11039308	A	G	0.014	0.002	1.28E-12	0.0295588	0.0295588	0.2	-	-	-
	rs1109088	A	G	0.013	0.002	2.60E-06	0.0198026	0.0198026	0.47	-	-	-
	rs1110698	A	C	-0.022	0.004	2.89E-05	-0.0833816	-0.0833816	0.0290001	-	-	-
	rs112613078	A	G	-0.025	0.003	1.55E-17	0.0676586	0.0676586	0.0140001	rs56186316	G	A
	rs11611435	T	C	0.012	0.002	4.00E-05	-0.0100503	-0.0100503	0.66	-	-	-

	rs11988076	A	G	-0.019	0.003	1.82E-08	0.0392207	0.0392207	0.18	-	-	-
	rs12025393	A	G	-0.014	0.002	1.82E-05	0.0198026	0.0198026	0.47	-	-	-
	rs12139650	T	G	0.015	0.003	2.21E-05	0.00995033	0.00995033	0.760001	rs11165789	G	T
	rs12140153	T	G	-0.027	0.004	4.62E-09	0.00995033	0.00995033	0.75	-	-	-
	rs12432176	A	C	0.013	0.002	4.02E-11	-0.0202027	-0.0202027	0.4	-	-	-
	rs12927162	A	G	0.028	0.002	2.11E-29	-0.0100503	-0.0100503	0.68	-	-	-
	rs12969848	T	C	0.017	0.002	1.52E-12	0.0295588	0.0295588	0.19	-	-	-
	rs13004345	T	C	-0.012	0.002	3.76E-05	-0.0202027	-0.0202027	0.34	-	-	-
	rs13306728	A	G	0.03	0.004	6.16E-11	0.0295588	0.0295588	0.4	-	-	-
	rs1421085	T	C	-0.023	0.002	1.39E-22	0.00995033	0.00995033	0.67	-	-	-
	rs17416934	T	C	0.013	0.002	4.02E-11	-0.0304592	-0.0304592	0.2	-	-	-
	rs17448682	T	C	0.018	0.003	4.04E-10	0.0392207	0.0392207	0.0700003	-	-	-
	rs1750785	A	G	0.014	0.002	3.52E-06	-0.0100503	-0.0100503	0.56	-	-	-
	rs17596722	T	C	-0.018	0.003	3.24E-08	0.00995033	0.00995033	0.69	-	-	-
	rs17604349	A	G	-0.022	0.003	1.21E-12	0.00995033	0.00995033	0.75	-	-	-
	rs1919346	A	G	0.012	0.002	1.30E-05	-0.0100503	-0.0100503	0.79	rs6468317	G	A
	rs2072727	T	C	0.012	0.002	3.46E-05	-0.0202027	-0.0202027	0.2	-	-	-
	rs2138759	A	G	0.014	0.002	1.75E-06	-0.0100503	-0.0100503	0.630001	-	-	-
	rs225289	T	C	0.016	0.003	1.24E-05	0.00995033	0.00995033	0.6	-	-	-
	rs2291589	T	G	0.018	0.002	1.65E-13	0.0295588	0.0295588	0.0920005	-	-	-
	rs2294203	A	G	0.013	0.002	2.97E-05	0.0198026	0.0198026	0.22	-	-	-
	rs2518022	T	C	0.032	0.004	1.53E-13	0.0392207	0.0392207	0.24	-	-	-
	rs2653349	A	G	0.031	0.003	1.03E-29	0.00995033	0.00995033	0.56	-	-	-
	rs2699869	A	C	0.013	0.002	5.06E-06	0.0198026	0.0198026	0.34	-	-	-
	rs2701524	T	C	0.013	0.002	5.39E-06	-0.0100503	-0.0100503	0.67	-	-	-

	rs2706762	T	C	-0.019	0.003	3.01E-07	-0.0618754	-0.0618754	0.0329997	-	-	-
	rs2712056	T	C	0.018	0.003	1.57E-07	-0.0202027	-0.0202027	0.31	-	-	-
	rs2737245	T	G	0.018	0.002	2.94E-11	-0.0100503	-0.0100503	0.81	rs4876611	T	G
	rs2794682	T	C	0.021	0.002	3.93E-19	-0.0202027	-0.0202027	0.41	-	-	-
	rs28634184	T	C	-0.014	0.002	2.31E-05	0.0198026	0.0198026	0.41	-	-	-
	rs286808	T	C	0.013	0.002	3.87E-06	0.0295588	0.0295588	0.14	-	-	-
	rs2881955	T	C	0.016	0.002	5.64E-08	0.0198026	0.0198026	0.32	-	-	-
	rs2910032	T	C	0.019	0.002	6.18E-15	0.00995033	0.00995033	0.61	-	-	-
	rs2916142	T	C	0.015	0.002	7.63E-09	-0.0100503	-0.0100503	0.48	-	-	-
	rs2971970	T	G	0.017	0.003	7.28E-09	0.0198026	0.0198026	0.3	-	-	-
	rs3100052	A	G	0.013	0.002	1.30E-05	-0.0304592	-0.0304592	0.11	-	-	-
	rs3168135	A	G	-0.017	0.003	3.64E-09	-0.0100503	-0.0100503	0.56	-	-	-
	rs335433	T	C	-0.013	0.002	2.29E-06	-0.0202027	-0.0202027	0.23	-	-	-
	rs35461065	T	C	-0.012	0.002	8.36E-06	0.0295588	0.0295588	0.15	-	-	-
	rs35748596	T	G	-0.016	0.002	2.37E-09	-0.0100503	-0.0100503	0.719999	rs4266671	G	T
	rs3797051	T	C	0.015	0.003	1.48E-06	-0.0100503	-0.0100503	0.58	-	-	-
	rs3843751	T	C	0.013	0.002	6.33E-06	-0.0618754	-0.0618754	0.000340001	-	-	-
	rs4102203	T	C	0.024	0.003	7.80E-09	0.0861777	0.0861777	0.00420001	-	-	-
	rs4237555	T	C	0.012	0.002	4.19E-05	-0.0304592	-0.0304592	0.15	-	-	-
	rs4241964	T	G	-0.015	0.002	3.54E-09	0.0392207	0.0392207	0.0719996	-	-	-
	rs4245555	T	C	-0.018	0.002	1.96E-12	0.0198026	0.0198026	0.22	-	-	-
	rs4557564	A	G	0.025	0.004	2.15E-06	0.0198026	0.0198026	0.67	-	-	-
	rs4672440	T	G	0.016	0.002	5.09E-10	0.0100503	0.0100503	0.75	rs778138	T	G
	rs486416	A	G	-0.013	0.002	6.07E-06	0.270027	0.270027	3.30E-55	-	-	-
	rs487952	A	G	0.013	0.002	4.43E-06	0.00995033	0.00995033	0.46	-	-	-

	rs4899502	A	G	-0.016	0.002	1.61E-08	0.00995033	0.00995033	0.51	-	-	-
	rs509476	T	C	0.099	0.006	7.41E-53	0.0295588	0.0295588	0.49	-	-	-
	rs56040212	A	G	-0.014	0.003	2.20E-05	0.0198026	0.0198026	0.31	-	-	-
	rs56049037	A	G	-0.017	0.002	2.28E-09	0.0295588	0.0295588	0.26	-	-	-
	rs56376592	A	C	0.017	0.003	1.40E-06	0.0295588	0.0295588	0.2	-	-	-
	rs56382918	T	C	0.018	0.002	1.13E-09	0.0100503	0.0100503	0.7	rs62448710	C	T
	rs57180764	A	G	0.017	0.003	3.22E-08	0.0198026	0.0198026	0.48	-	-	-
	rs605765	T	C	-0.012	0.002	3.13E-05	-0.0304592	-0.0304592	0.19	-	-	-
	rs61773390	T	G	0.029	0.003	2.75E-24	0.0582689	0.0582689	0.005	-	-	-
	rs61963123	T	C	0.017	0.003	2.33E-07	-0.0304592	-0.0304592	0.2	-	-	-
	rs62172117	A	G	-0.019	0.002	1.43E-14	-0.0100503	-0.0100503	0.6	-	-	-
	rs62182135	A	C	-0.013	0.002	7.80E-06	0.0100503	0.0100503	0.75	rs55962118	A	C
	rs62263597	A	G	0.028	0.004	4.79E-10	-0.0202027	-0.0202027	0.5	-	-	-
	rs6472936	T	C	0.018	0.003	5.16E-09	0.00995033	0.00995033	0.780001	-	-	-
	rs6504758	A	G	-0.014	0.002	9.83E-08	-0.00995033	-0.00995033	0.69	rs2202237	A	G
	rs671255	A	G	-0.014	0.002	1.42E-05	0.0295588	0.0295588	0.13	-	-	-
	rs6716898	A	G	0.021	0.002	2.32E-19	0.0676586	0.0676586	0.000560003	-	-	-
	rs6769642	A	C	0.016	0.002	2.80E-10	-0.0100503	-0.0100503	0.39	-	-	-
	rs6778003	T	G	0.016	0.002	4.94E-09	0.00995033	0.00995033	0.81	-	-	-
	rs6799356	A	C	0.014	0.003	1.53E-06	0.0198026	0.0198026	0.54	-	-	-
	rs6935086	T	C	0.02	0.003	1.31E-11	0.122218	0.122218	0.02	-	-	-
	rs695459	T	C	-0.013	0.002	6.59E-06	-0.0100503	-0.0100503	0.79	-	-	-
	rs7138306	A	G	0.013	0.002	4.02E-11	0.0198026	0.0198026	0.36	-	-	-
	rs7196720	T	C	0.013	0.002	4.32E-06	-0.0100503	-0.0100503	0.46	-	-	-
	rs72720396	A	G	-0.022	0.003	3.29E-15	-0.0304592	-0.0304592	0.14	-	-	-

	rs7313852	A	G	-0.025	0.002	1.02E-26	-0.0202027	-0.0202027	0.35	-	-	-
	rs7337911	A	G	0.015	0.002	1.00E-06	0.00995033	0.00995033	0.59	-	-	-
	rs7428484	A	G	0.013	0.002	1.97E-05	-0.0100503	-0.0100503	0.57	-	-	-
	rs7488974	A	G	0.016	0.002	1.81E-10	0.0100503	0.0100503	0.8	rs7959983	A	G
	rs7579662	A	G	-0.012	0.002	2.24E-05	-0.0304592	-0.0304592	0.18	-	-	-
	rs76518095	T	C	0.023	0.004	1.00E-05	-0.00995033	-0.00995033	0.79	rs7086182	T	C
	rs77598468	A	C	-0.042	0.006	1.05E-09	0.040822	0.040822	0.43	rs62550819	A	C
	rs78095690	T	C	-0.013	0.002	5.75E-06	0.0202027	0.0202027	0.28	rs6043841	C	T
	rs827751	A	G	0.019	0.002	1.05E-21	-0.0512933	-0.0512933	0.0219999	-	-	-
	rs925947	T	G	0.016	0.003	9.64E-07	0.0198026	0.0198026	0.47	rs6265	T	G
	rs9295795	T	C	-0.026	0.005	1.90E-05	0.19062	0.19062	4.70E-08	-	-	-
	rs9348050	T	C	0.013	0.002	2.00E-06	-0.0100503	-0.0100503	0.59	-	-	-
	rs9395520	T	C	0.02	0.002	1.60E-15	-0.0100503	-0.0100503	0.51	-	-	-
	rs9416744	A	C	0.017	0.002	1.47E-09	0.0198026	0.0198026	0.33	-	-	-
	rs9521184	T	C	0.013	0.002	8.34E-07	0.00995033	0.00995033	0.62	-	-	-
	rs9565309	T	C	0.066	0.006	1.26E-25	0.0487902	0.0487902	0.38	-	-	-
	rs9597250	A	C	-0.019	0.003	2.88E-09	-0.0304592	-0.0304592	0.16	-	-	-
	rs9636202	A	G	-0.013	0.002	3.53E-05	-0.0304592	-0.0304592	0.0959997	-	-	-
	rs974552	A	G	-0.022	0.003	1.40E-12	-0.00995033	-0.00995033	0.760001	rs6506291	G	A
	rs9795439	A	G	0.018	0.003	9.87E-10	-0.0202027	-0.0202027	0.47	-	-	-
	rs9898091	T	C	0.038	0.006	6.88E-09	0.113329	0.113329	0.0870001	-	-	-
	rs9964420	A	C	-0.022	0.002	4.03E-18	0.0198026	0.0198026	0.38	-	-	-
snoring	rs10062026	A	G	-0.030459207	0.005204	2.08E-08	0.0487902	0.0194217	0.012	-	-	-
	rs1016013	A	G	-0.030459207	0.005145	7.22E-10	-0.0202027	0.0207396	0.33	-	-	-
	rs10878269	T	C	0.039220713	0.005176	3.76E-13	0.00995033	0.0226189	0.66	-	-	-

	rs12449843	A	G	-0.030459207	0.005151	4.45E-10	-0.0304592	0.021678	0.16	-	-	-
	rs1641511	G	A	0.029558802	0.005956	1.40E-08	0.0100503	0.0235841	0.67	-	-	-
	rs17060460	G	A	0.029558802	0.005955	1.43E-08	-0.0198026	0.0207539	0.34	-	-	-
	rs2307111	C	T	-0.030459207	0.005108	1.34E-11	-0.00995033	0.0140879	0.48	-	-	-
	rs2664299	C	T	-0.030459207	0.005067	4.48E-11	-0.00995033	0.0212751	0.64	-	-	-
	rs2981329	C	T	0.029558802	0.00579	7.74E-09	-0.0295588	0.0230649	0.2	-	-	-
	rs34107769	C	T	-0.030459207	0.005485	9.93E-09	-0.0198026	0.028037	0.48	-	-	-
	rs35562935	A	G	0.058268908	0.009454	1.69E-08	0.0392207	0.037842	0.3	-	-	-
	rs4987719	T	C	0.076961041	0.01368	3.59E-08	0.207014	0.120575	0.0860003	-	-	-
	rs57222984	G	A	0.039220713	0.005824	3.03E-11	-0.0100503	0.0228462	0.66	rs62055714	G	A
	rs592333	G	A	0.039220713	0.005012	1.08E-16	-0.00995033	0.0219285	0.649999	-	-	-
	rs6855873	T	C	0.029558802	0.005822	2.08E-08	-0.0304592	0.021678	0.16	-	-	-
	rs7007887	T	C	0.039220713	0.005024	9.88E-14	-0.0100503	0.0214889	0.64	-	-	-
	rs732172	T	C	0.029558802	0.005154	1.35E-09	0.00995033	0.0184663	0.59	-	-	-
	rs79275904	T	C	-0.051293294	0.008855	5.64E-09	0.0295588	0.0374545	0.43	rs7221192	T	C
	rs8047587	T	G	0.029558802	0.005036	2.13E-09	-0.0100503	0.0243666	0.68	rs11642015	T	G
	rs9309771	A	G	0.029558802	0.005018	5.65E-09	-0.0512933	0.01787	0.00409996	-	-	-
	rs947612	G	A	0.029558802	0.005761	9.92E-09	0.0100503	0.0168133	0.55	-	-	-
	rs9933881	C	T	0.058268908	0.00948	8.94E-09	0.0512933	0.033877	0.13	-	-	-

FigureS1. Sensitivity analysis (A), scatter plot (B), forest plot (C), and funnel plot (D) of the causal effect of frequent insomnia on RA risk.

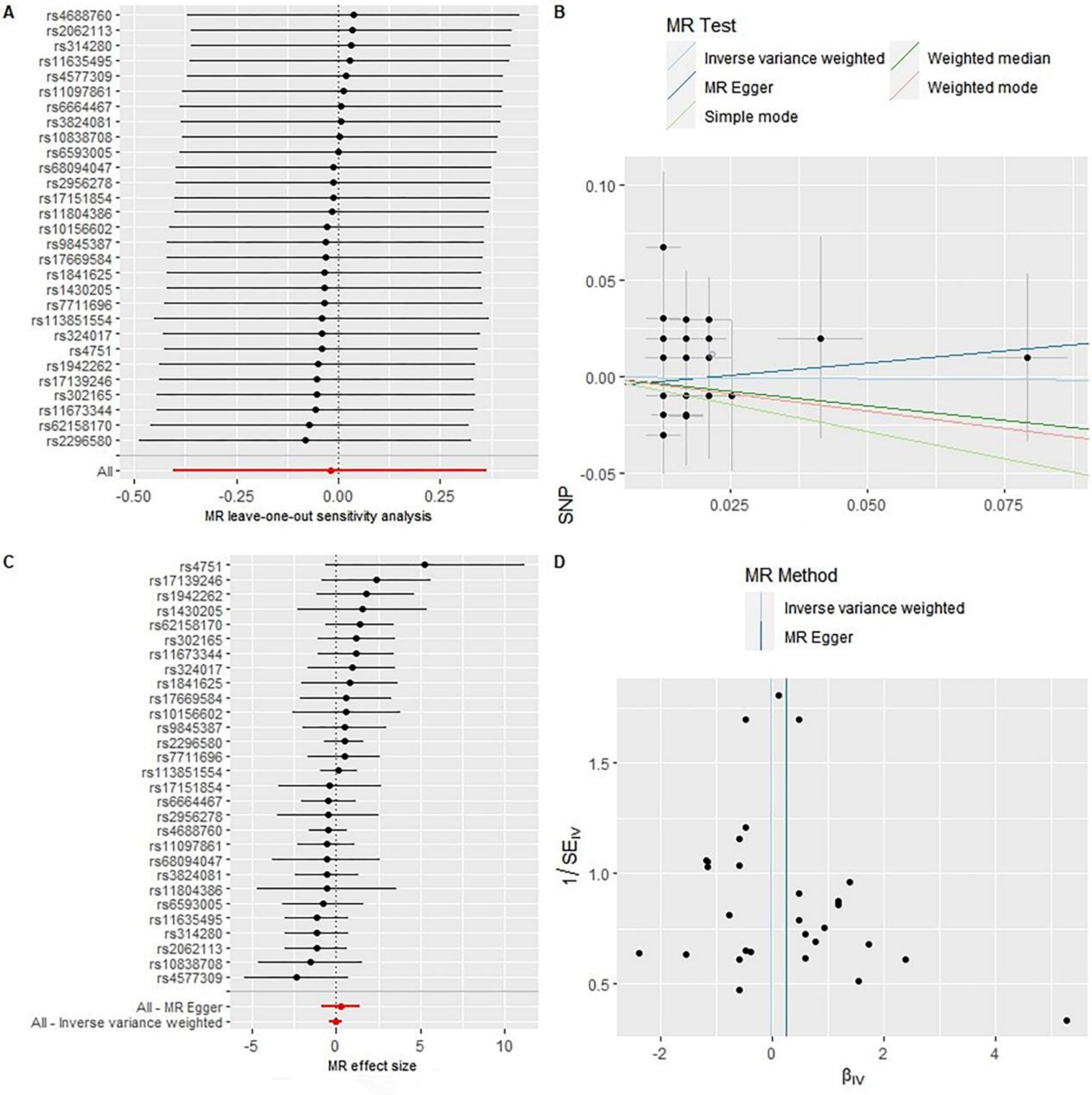

FigureS2. Sensitivity analysis (A), scatter plot (B), forest plot (C), and funnel plot (D) of the causal effect of any insomnia on RA risk.

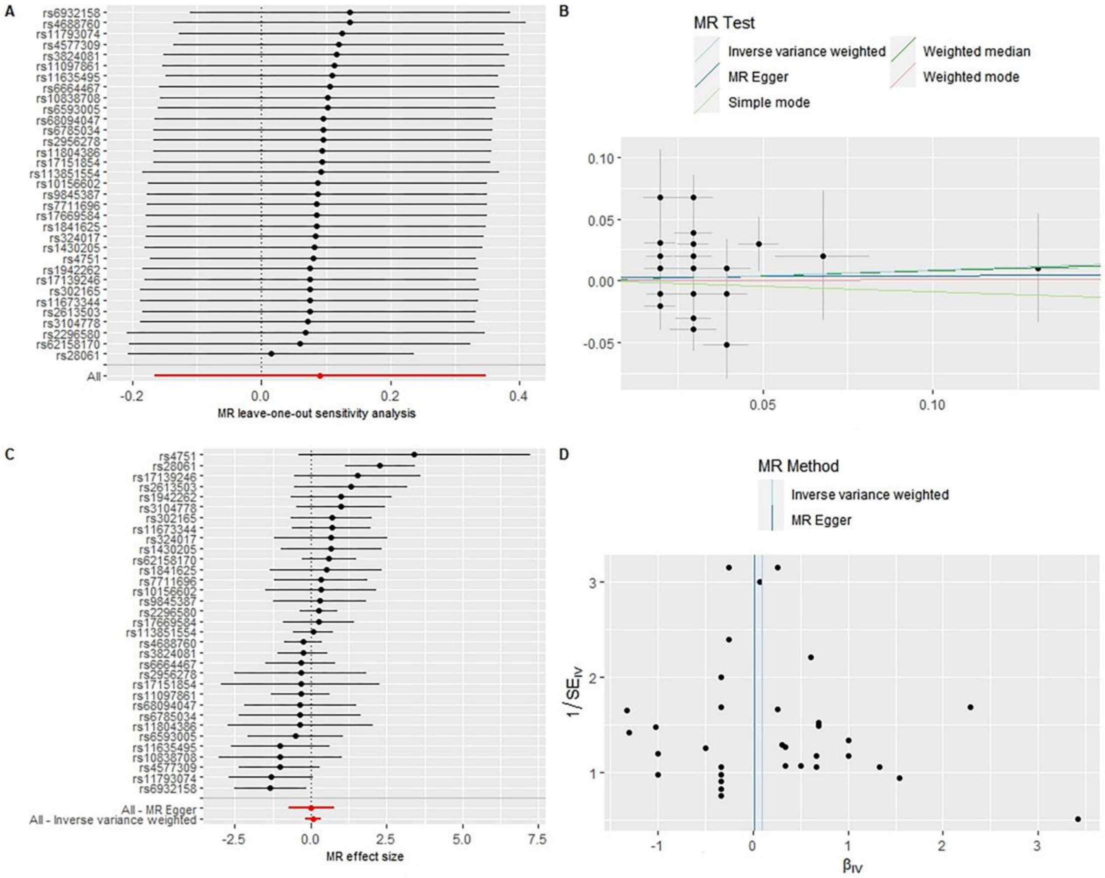

FigureS3. Sensitivity analysis (A), scatter plot (B), forest plot (C), and funnel plot (D) of the causal effect of sleep duration on RA risk.

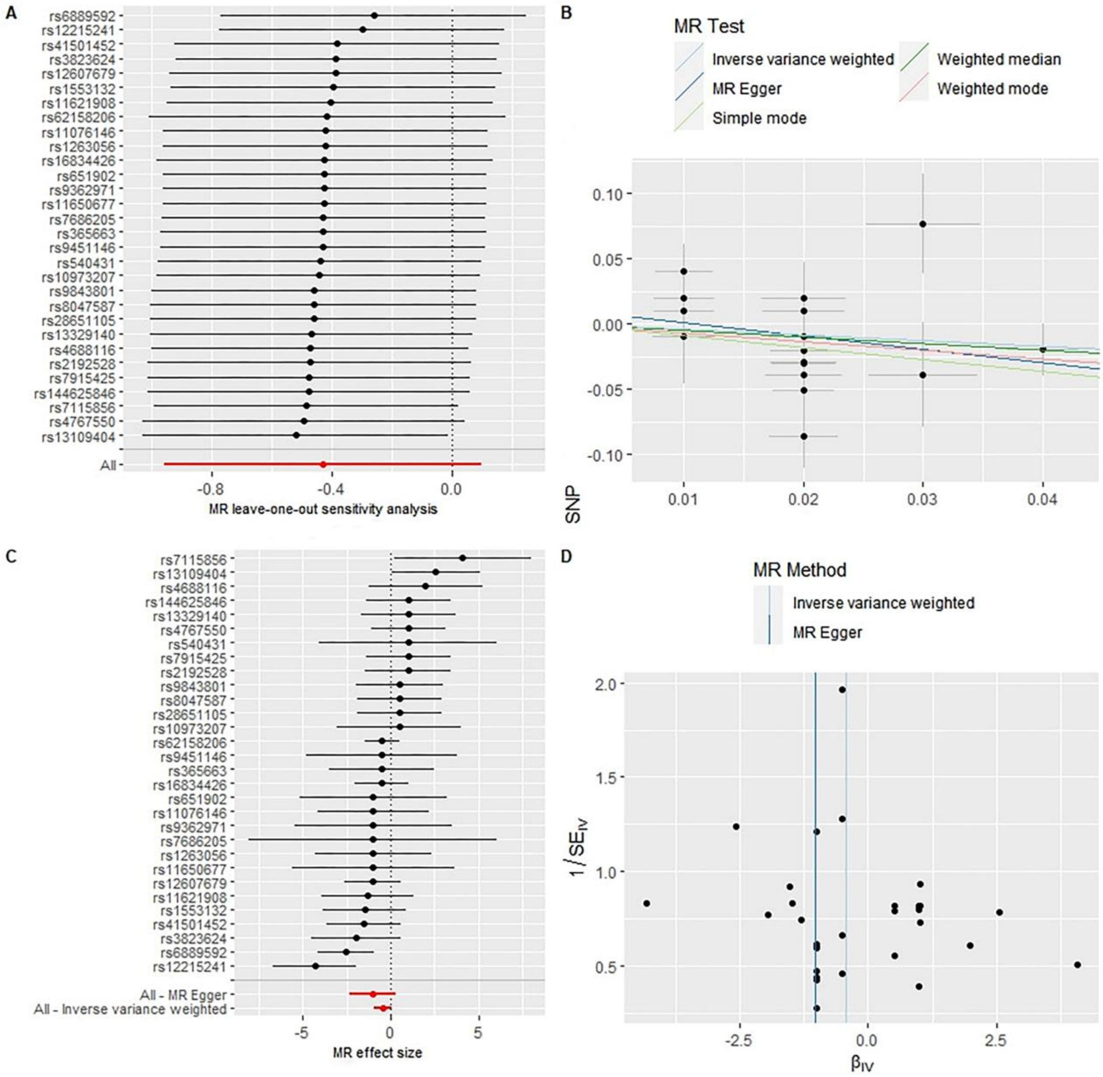

FigureS4. Sensitivity analysis (A), scatter plot (B), forest plot (C), and funnel plot (D) of the causal effect of getting up on RA risk.

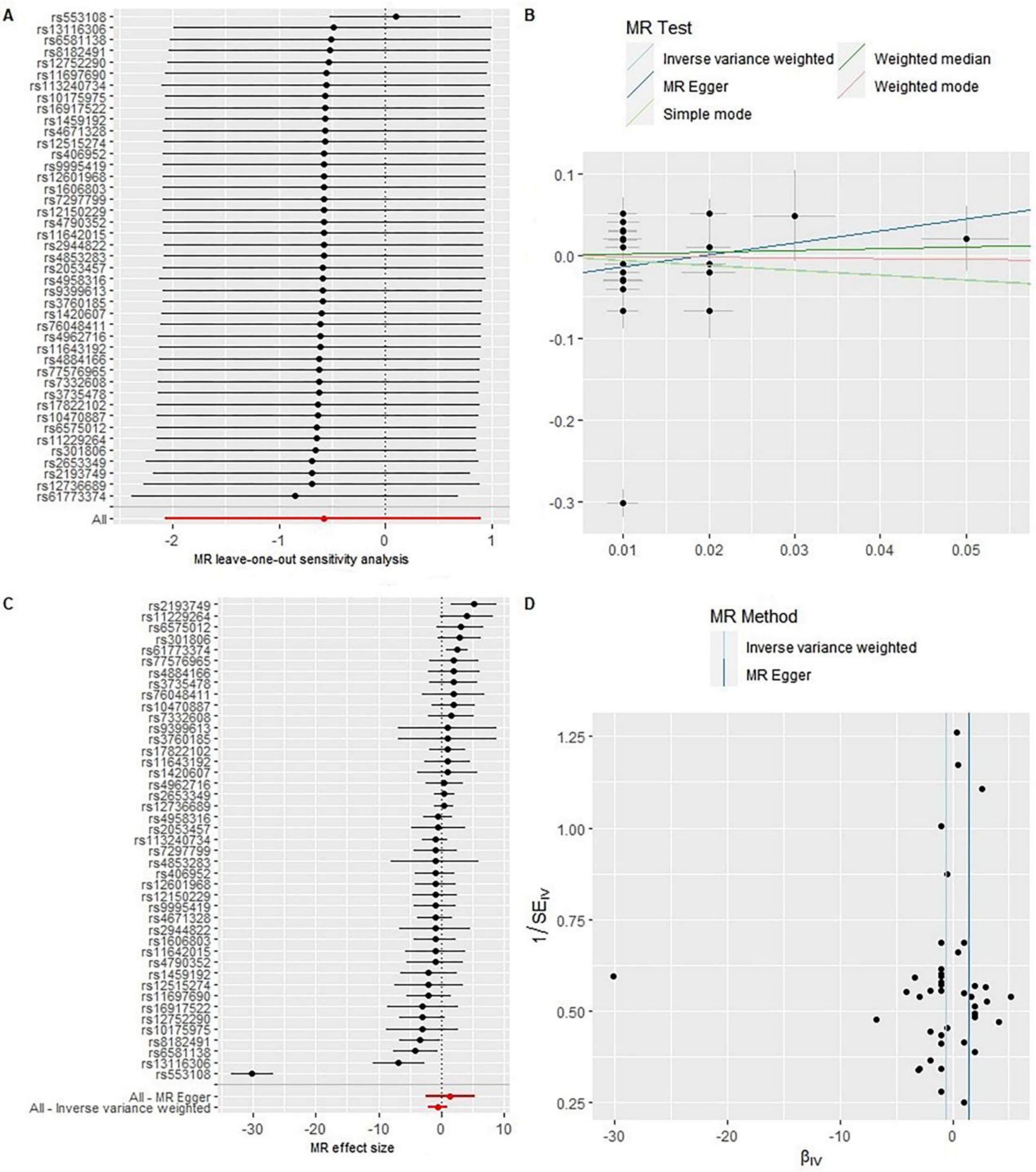

FigureS5. Sensitivity analysis (A), scatter plot (B), forest plot (C), and funnel plot (D) of the causal effect of morningness on RA risk.

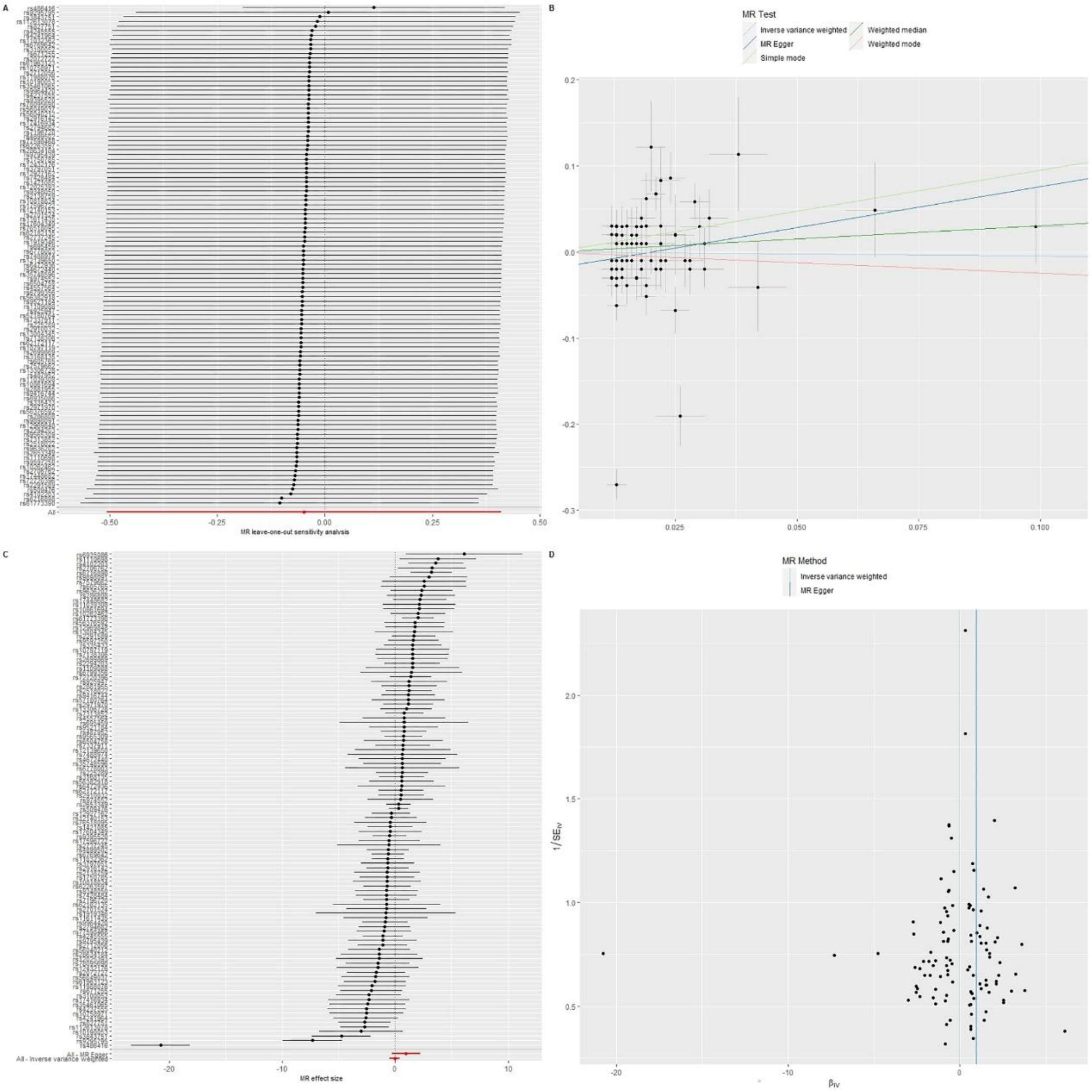

FigureS6. Sensitivity analysis (A), scatter plot (B), forest plot (C), and funnel plot (D) of the causal effect of snoring on RA risk.

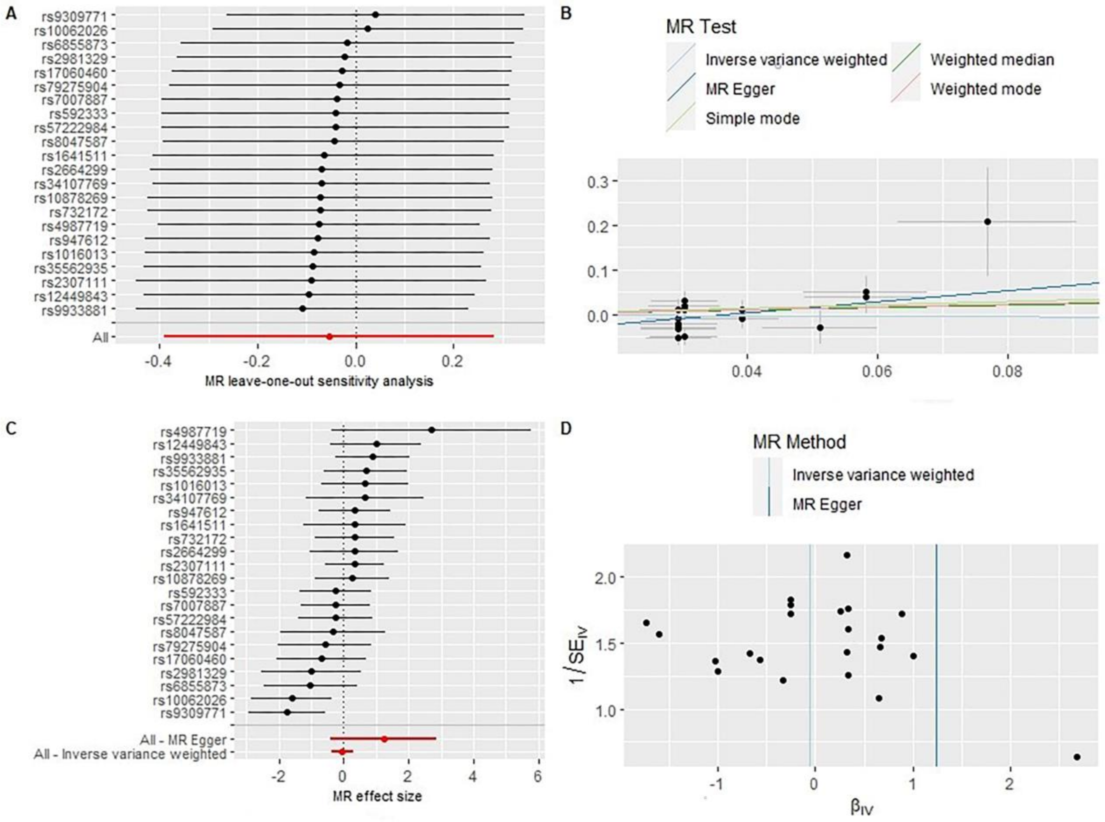

Supplement: Supplementary file 1 [file Data_Sheet_1.pdf]
